# Efficacy of lopinavir–ritonavir combination therapy for the treatment of hospitalized COVID-19 patients: a meta-analysis

**DOI:** 10.2217/fvl-2021-0066

**Published:** 2022-01-31

**Authors:** Jiawen Deng, Fangwen Zhou, Wenteng Hou, Kiyan Heybati, Saif Ali, Oswin Chang, Zachary Silver, Thanansayan Dhivagaran, Harikrishnaa Ba Ramaraju, Chi Yi Wong, Qi Kang Zuo, Elizabeth Lapshina, Madeline Mellett

**Affiliations:** ^1^Faculty of Health Sciences, McMaster University, 1280 Main St W, Hamilton, ON, L8S 4L8, Canada; ^2^Mayo Clinic Alix School of Medicine, Mayo Clinic, 200 First St SW, Rochester, MN 55905, USA; ^3^Faculty of Science, Carleton University, 1125 Colonel By Dr, Ottawa, ON, K1S 5B6, Canada; ^4^Integrated Biomedical Engineering & Health Sciences Program, McMaster University, 1280 Main St W, Hamilton, ON, L8S 4L8, Canada; ^5^Department of Anesthesiology, Rutgers, New Jersey Medical School, 185 S Orange Ave, Newark, NJ 07103, USA; ^6^Faculty of Science, McGill University, 845 Sherbrooke St W, Montreal, QC, H3A 0G4, Canada

**Keywords:** adverse events, antiviral, covid-19, lopinavir, mortality, ritonavir, SARS-CoV-2

## Abstract

**Aim:** To evaluate the efficacy and safety of lopinavir–ritonavir (LPV/r) therapy in treating hospitalized COVID-19 patients. **Materials & methods:** Data from randomized and observational studies were included in meta-analyses. Primary outcomes were length of stay, time for SARS-CoV-2 test conversion, mortality, incidence of mechanical ventilation, time to body temperature normalization and incidence of adverse events. **Results:** Twenty-four studies (n = 10,718) were included. LPV/r demonstrated no significant benefit over the control groups in all efficacy outcomes. The use of LPV/r was associated with a significant increase in the odds of adverse events. **Conclusion:** Given the lack of efficacy and increased incidence of adverse events, the clinical use of LPV/r in hospitalized COVID-19 patients is not recommended.

In December 2019, a series of acute, atypical cases of pneumonia, characterized by its rapid rate of transmission, was identified in Wuhan, China. The source of the illness was quickly attributed to a new strain of coronavirus, named SARS-CoV-2, and the subsequent disease it caused was dubbed COVID-19 [1,2]. Since the WHO designated COVID-19 as a global pandemic in March 2020 [3,4], researchers around the world have worked tirelessly to identify effective treatment strategies and design vaccines to treat millions of infected patients and reduce the rate of SARS-CoV-2 infections.

Despite the early success of vaccines from Pfizer & BioNTech [5], AstraZeneca [6] and Moderna [7], various obstacles have slowed the manufacturing and distribution of SARS-CoV-2 vaccines [8,9]. Due to these challenges and the drastic increase in COVID-19 cases during the first quarter of 2021 [10], finding an effective treatment regimen for COVID-19 has never been more important. As of 1 March 2020, there were over 5000 registered COVID-19-related clinical trials that are either recruiting, ongoing or completed [11]. However, consensus regarding the clinical management of COVID-19 is still contradictory and unclear [12,13], especially surrounding popular and controversial regimens such as hydroxychloroquine [14,15]. Currently, treatment strategies for combating COVID-19 is largely based on evidence-based guidelines involving repurposed antiviral therapies, such as remdesivir [16], and immunosuppressive drugs, such as corticosteroids (i.e., dexamethasone) [17,18].

Lopinavir–ritonavir (LPV/r) is a protease inhibitor combination used for the treatment of human immunodeficiency virus (HIV) and was also repurposed as a potential antiviral therapy for the treatment of COVID-19 [19]. Initial enthusiasm regarding the efficacy of LPV/r was largely due to its ability to prevent cytotoxicity and reduce viral load *in vitro* [20], as well as encouraging *in vivo* evidence suggesting that LPV/r may have been effective against the severe acute respiratory syndrome (SARS) with low incidences of adverse events in 2004 [21–23]. Both lopinavir and ritonavir are competitive inhibitors of viral proteases which prevents the post-translational proteolysis of precursor peptides and the subsequent release of functional viral proteins, resulting in the production of immature viral particles [24,25]. They are commonly used in combination due to the low oral bioavailability of lopinavir, which requires ritonavir as a booster to achieve therapeutic drug concentration [26,27]. It is postulated that LPV/r may bind to the highly conserved substrate-binding pocket region of the 3C-like proteinase (3CL^pro^) of coronaviruses, leading to its anti-coronavirus capabilities [28,29]. *In silico* binding studies involving LPV/r showed that LPV/r is capable of binding to the SARS coronavirus (SARS-CoV) proteases, and it is speculated that LPV/r should be able to bind to the 3CL^pro^ protein of SARS-CoV-2 as well due to the highly conserved nature of the 3CL^pro^ substrate binding site between SARS-CoV and SARS-CoV-2 [30,31].

Current evidence regarding the clinical use of LPV/r is conflicting and of low quality. Several open-labeled international randomized controlled trials (RCTs), including the RECOVERY trial [32], the WHO SOLIDARITY trial [33] and an RCT reported by Cao *et al.* [34], have all reported a lack of benefits associated with LPV/r treatments in regards to mortality, viral clearance and time to clinical improvements. However, non-randomized observational studies [35–39] have often produced more optimistic results, suggesting that LPV/r may reduce time to viral clearance and viral shedding. The use of LPV/r for treating COVID-19 is still recommended in several countries, including China (in combination with interferon) [40], Egypt, Saudi Arabia, Belgium and Ireland [41], suggesting that the potential efficacy of LPV/r is still recognized, or at least, debated, by national health organizations. Conflicting opinions on the treatment of COVID-19 from medical guidelines could result in public mistrust, further exacerbating potential issues such as vaccine hesitancy [42,43].

Previous systematic reviews and meta-analyses regarding the LPV/r usage for treating COVID-19 patients yielded no statistically significant difference between LPV/r and standard of care in multiple patient-important outcomes, such as mortality, disease progression and length of stay [44,45]. However, these early reviews often included a small number of trials with low sample sizes, which may not be able to provide the necessary precision to detect significant treatment effects [46]. Most evidence-based guidelines were also based upon major RCTs such as the RECOVERY and SOLIDARITY trials, while not accounting for observational evidence. Lastly, many systematic reviews involving COVID-19 failed to account for data from non-English databases, namely Chinese databases, which may contain many unanalyzed trials due to the large number of COVID-19 cases in China during the early stages of the pandemic [47,48]. Given the limitations of previous knowledge synthesis studies, we conducted an updated systematic review and meta-analysis to investigate whether the use of LPV/r, with or without adjuvant therapies, is more beneficial compared with standard of care or adjuvant therapies alone in regards to length of stay, time for positive-to-negative conversion of SARS-CoV-2 nucleic acid tests and mortality in hospitalized patients with COVID-19. We also examined the safety of LPV/r therapy in COVID-19 patients, including incidences of adverse events and severe (grade 3/4) adverse events.

## Methods

We conducted this systematic review and meta-analysis following recommendations from the Cochrane Handbook for Systematic Reviews of Interventions [49] and in accordance to the PRISMA statements [50]. See online Supplementary Table 1 for the completed PRISMA checklist. This review was prospectively registered on PROSPERO (CRD42021241183), the international prospective register of systematic reviews [51].

### Study identification

We searched the following databases from 1 January 2020 to 10 February 2021 using English search strategies consisting of the keywords ‘lopinavir’, ‘ritonavir’, ‘lopinavir–ritonavir’, ‘LPV’, ‘Norvir’ and ‘Kaletra’ in combination with database-specific COVID-19 search strings provided by the Rudolph Matas Library of the Health Sciences of Tulane University [52]: Medical Literature Analysis and Retrieval System Online (MEDLINE), Excerpta Medica Database (EMBASE) and PubMed. Additionally, we systematically searched the following Chinese databases from 1 January 2020 to 10 February 2021 using a custom Chinese search strategy: Wanfang Data, Wanfang Med Online, SinoMed, China National Knowledge Infrastructure (CNKI) and Chongqing VIP Information (CQVIP). The search strategies used for the database searches can be found in online Supplementary Tables 2–9. We did not impose language restrictions during our study identification and selection processes.

Due to potential biased or problematic COVID-19 studies being published on preprint repositories [53–55], we opted to limit our literature search to peer-reviewed sources only. We also hand-searched the reference sections of two previous systematic reviews [44,45] for relevant studies that were not identified by our database searches.

### Eligibility criteria

We included both RCTs and comparative non-randomized observational studies that satisfied the following inclusion criteria in our analysis: compared LPV/r with standard of care, or compared LPV/r with adjuvant therapies to adjuvant therapies alone, included laboratory-confirmed, hospitalized COVID-19 patients. While we included studies involving LPV/r with adjuvant therapies, we only included studies that used the same concurrent therapy for its intervention and control arms to minimize the effect of adjuvant therapies on treatment outcomes, similar to the design of several other meta-analyses [56–58].

### Outcome measures

Our primary outcomes include: length of stay, time for positive-to-negative SARS-CoV-2 nucleic acid tests, mortality at the latest follow-up, incidence of mechanical ventilation, time to normalization of body temperature and incidence of adverse events. Our secondary outcomes include: rate of positive-to-negative conversion at day 7 and day 14, and incidence of severe (grade 3/4) adverse events.

### Study selection

**Figure 1.**
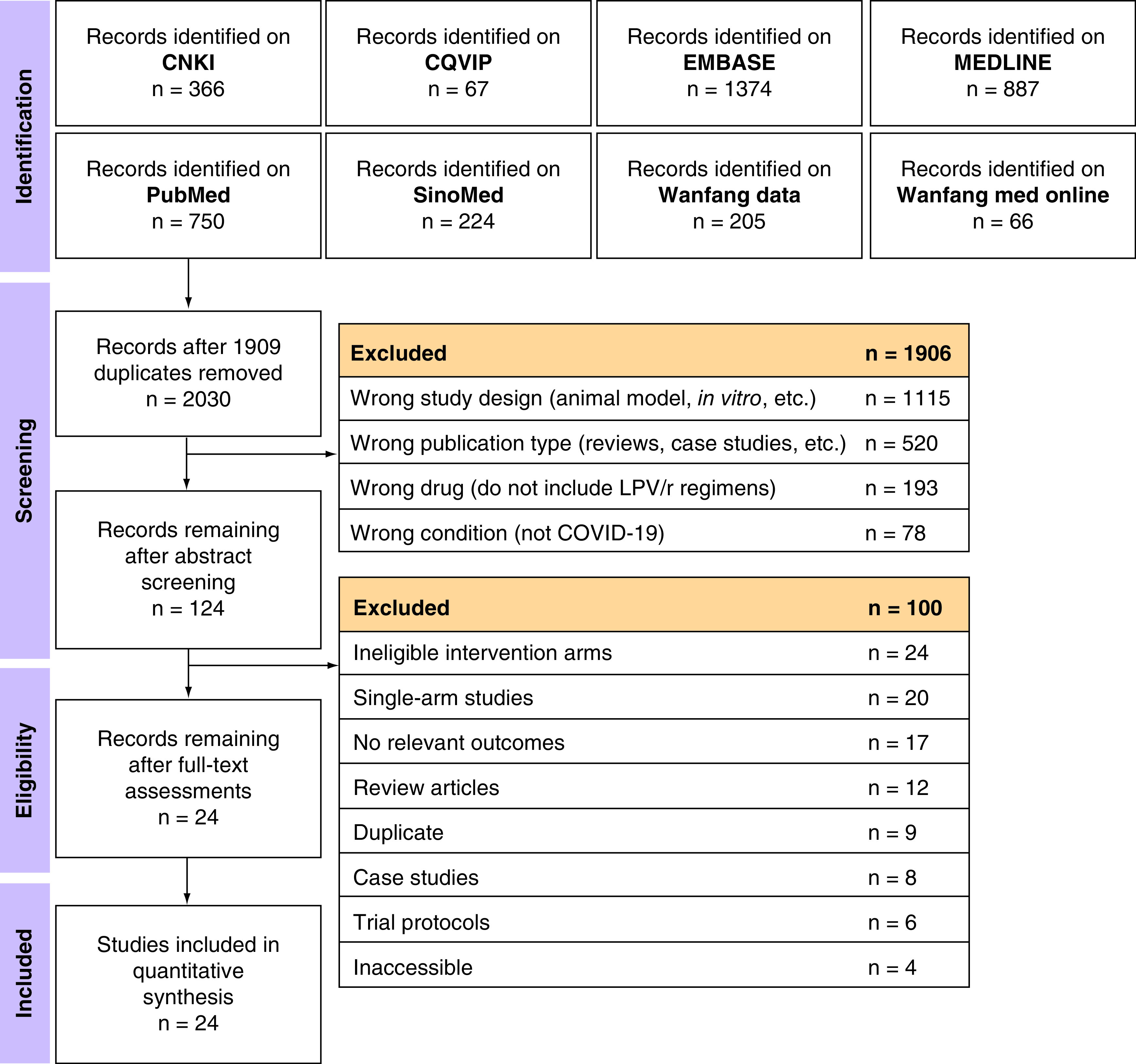
PRISMA flowchart for the identification and selection of studies. CNKI: China National Knowledge Infrastructure; CQVIP: Chongqing VIP Information; EMBASE: Excerpta Medica Database; LPV/r: Lopinavir–ritonavir; MEDLINE: Medical Literature Analysis and Retrieval System Online.

We performed title and abstract screening using Rayyan (https://rayyan.qcri.org/) [59] independently and in duplicate based on the eligibility criteria. Included abstracts were entered into an independent and in duplicate full text screening process. We resolved disagreements by recruiting a senior author to attain consensus. Figure 1 shows the PRISMA flowchart [60] of our study selection process.

### Data extraction

We performed data extraction independently and in duplicate using extraction sheets developed *a priori*. We extracted information relating to baseline demographics, descriptions of study methodology, treatment descriptions and outcome measures. The full list of extracted items can be found on our PROSPERO registration.

### Risk of bias assessment

We assessed the risk of bias in the included RCTs using the revised Cochrane risk of bias tool for randomized trials (RoB2) [61]. The risk of bias in non-randomized observational studies was assessed using the risk of bias in non-randomized studies of interventions (ROBINS-I) [62]. Reviewers judged the risk of bias in each RoB2 or ROBINS-I domain independently and in duplicate, and resolved disagreements by consulting with a senior author.

### Quality of evidence

We assessed the quality of evidence for our primary outcomes using the Grading of Recommendations, Assessment, Development and Evaluations (GRADE) framework [63,64]. The GRADE approach evaluates the quality of evidence by assessing the following domains: study limitations (risk of bias) [65], indirectness [66], inconsistency [67], imprecision [68] and publication bias [69]. Quality of evidence may also be rated up due to magnitude of effects, dose-response gradients and plausible confounders [70]. The overall quality of evidence for each outcome was rated as either high, moderate, low or very low [71], and the results of the primary outcomes and GRADE ratings were presented in a GRADE summary of findings table [72] generated using the GRADEpro online software (https://gradepro.org/) [73].

### Statistical analysis

We conducted all statistical analyses using R 4.0.4 (https://www.r-project.org/) [74], and we performed random-effects meta-analyses using the *meta* 4.18 library (https://cran.r-project.org/web/packages/meta/) [75]. We expressed and pooled the treatment effects of dichotomous outcomes as odds ratios (ORs) and 95% confidence intervals (CIs), and we also calculated the number needed to treat (NNT) and harm (NNH) [76] for dichotomous outcomes. We reported the treatment effect of continuous outcomes as mean differences (MDs) and 95% CIs.

#### Missing data & rare events

For studies with missing data required for analysis, including the mean outcome value and measure of variance for continuous outcomes, attempts were made to contact corresponding authors to obtain unpublished data. For studies that presented continuous outcomes in median and interquartile range (IQR), we assumed that the outcome data is normally distributed and used methods recommended by Luo *et al.* [77] and Wan *et al.* [78] to estimate the mean and standard deviation (SD) for analysis. We tested the impact of this assumption by conducting a subgroup analysis comparing the pooled results from studies with estimated mean and SD to studies that did not require estimation.

For studies reporting zero events in one of its treatment arms, we applied a continuity correction factor of 0.5 [79] to complete the meta-analysis. We did not include studies that reported zero events in all treatment arms in our analyses.

#### Heterogeneity assessment

We assessed the presence of heterogeneity using the Cochran's Q test [80] with a significance level of p < 0.10, as recommended by the Cochrane Handbook [49]. Heterogeneity was quantified using I^2^ statistics [80,81]. We interpreted 30% < I^2^ < 75% as moderate heterogeneity and I^2^ ≥ 75% as serious heterogeneity [49].

#### Publication bias

We drew funnel plots [49] to identify small study effects within our included studies as a signal for the presence of publication bias, and we used Egger's regression test to quantitatively evaluate asymmetry within the funnel plots [82]. Egger's regression test was not conducted if fewer than 10 studies were included in the analysis, as the test may lack power in these circumstances [83]. We used the trim-and-fill method [84,85] to estimate the number of missing, unpublished studies and to observe the impact of unpublished studies on the pooled treatment effect when potential publication bias is detected.

#### Meta-regression & subgroup analysis

We performed meta-regression analyses on the proportion of patients with severe disease, defined according to individual study criteria. We performed subgroup analyses based on factors defined *a priori*: study design (randomized vs nonrandomized), adjuvant regimens and daily LPV/r dosage. For the outcome of mortality, we also conducted a meta-regression to examine the impact of different follow-up durations on the treatment effect. We planned to conduct subgroup analyses based on risk of bias rating (low/some concerns RoB2 + low/moderate ROBINS-I vs high RoB2 + serious/critical ROBINS-I). However, this was not completed as only observational studies were included in the high risk subgroup, and only RCTs were included in the low risk subgroup. This observation makes the subgroup analysis by risk of bias rating redundant.

## Results

### Included studies

We identified and screened 2030 (after deduplication using Endnote 20 [https://endnote.com/]) potentially eligible titles/abstracts. One hundred and twenty four full texts were retrieved and screened. Four RCTs [32–34,86] and 20 observational studies [36–38,87–103] were ultimately included in the systematic review and meta-analysis (Figure 1) with a total of 10,718 hospitalized COVID-19 patients. Characteristics of included studies are tabulated in Table 1. Four studies [89–91,95] only included patients hospitalized in intensive care units (ICUs), while Yao *et al.* [100] excluded ICU-hospitalized patients. Cao *et al.* [34] and Lecronier *et al.* [91] only included patients with severe disease, while four studies [86,88,92,98] excluded severe patients.

**Table 1.** Characteristics of included studies and patients.

Study (year)	Design	Country	Treatment arms	Treatment description	Sample size (n)	Patients with severe disease (n)	F/M	Age (years)^†^	Treatment duration (days)	Ref.
Cao *et al.* (2020)	Parallel RCT	China	LPV/r	0.4 g LPV + 0.1 g RTV, b.i.d.	99	99	38/61	58 (50–68)	14	[34]
			Standard of Care	Antibiotics, vasopressor, renal-replacement therapy, supplemental oxygenation and ventilation	100	100	41/59	58 (48–68)	–	
Echarte-Morales *et al.* (2021)	Prospective cohort	Spain	LPV/r + HCQ + AZM	HCQ + AZM, 0.2 g LPV + 0.5 g RTV b.i.d.	114	–	48/66	69 (18)	14	[93]
			HCQ + AZM	HCQ 400 mg b.i.d. on day 1, 200 mg b.i.d. on day 2–5 + AZM 500 mg q.d. on day 1, 250 mg q.d. on day 2–5	54	–	22/32	65 (13)	5	
Gao *et al.* (2020)	Retrospective cohort	China	LPV/r	LPV/r 500 mg, b.i.d.	51	0	21/30	33 (27–41)	7	[88]
			Standard of care	–	59	0	29/30	30 (23–45)	–	
Grimaldi *et al.* (2020)	Retrospective cohort	France/Belgium	LPV/r	–	57	–	11/46	63 (12)	–	[90]
			Standard of care	–	85	–	21/64	63 (11)	–	
Hraiech *et al.* (2020)	Retrospective cohort	France	LPV/r	LPV/r 800 mg q.d.	13	–	4/9	62 (13)	–	[95]
			Standard of care	–	15	–	4/11	60 (16)	–	
Chen *et al.* (2020)	Retrospective cohort	China	LPV/r + IFN-α2b	IFN-α2b, 0.2 g LPV + 0.05 g RTV x2 b.i.d.	52	0	25/27	47 (35–60)	5	[102]
			IFN-α2b	–	48	4	24/24	55 (36–62)	5	
Wang *et al.* (2020)	Retrospective cohort	China	LPV/r	0.2 g LPV+ 0.05 g RTV x2 b.i.d.	34	34	9/25	66 (53–83)	LOS	[103]
			Standard of care	–	22	22	5/17	76 (71–86)	LOS	
Karolyi *et al.* (2020)	Prospective cohort	Austria	LPV/r	0.4 g LPV + 0.1 g RTV b.i.d.	47	–	15/32	65 (49–72)	7	[94]
			Standard of care	–	89	–	43/46	77 (60–81)	–	
Lecronier *et al.* (2020)	Retrospective cohort	France	LPV/r	LPV/r 400 mg b.i.d.	20	20	5/15	55 (49–61)	4	[91]
			Standard of care	–	22	22	4/18	63 (54–70)	–	
Levy *et al.* (2020)	Retrospective cohort	France	LPV/r	0.4 g LPV + 0.1 g RTV b.i.d.	12	–	5/7	61 (54–73)	14	[89]
			Standard of care	–	30	–	10/20	64 (53–69)	–	
Li *et al.* (2020)	Parallel RCT	China	LPV/r	0.2 g LPV + 0.05 g RTV x2 b.i.d.	34	0	17/17	50 (15)	7–14	[86]
			Standard of care	–	17	0	10/7	44 (13)	7–14	
Liu *et al.* (2020)	Retrospective cohort	China	LPV/r + IFN	IFN, LPV/r 500 mg b.i.d.	65	–	33/32	37 (14)	–	[97]
			IFN	IFN 5 MU b.i.d.	37	–	16/21	37 (33)	–	
Wang *et al.* (2020)	Retrospective cohort	China	LPV/r + IFN	–	83	24	28/55	53 (42–62)	–	[96]
			IFN	–	39	10	23/16	52 (41–56)	–	
Nathalie *et al.* (2020)	Retrospective cohort	Switzerland	LPV/r + HCQ	LPV/r, HCQ	158	0	101/57	62 (15)	5	[92]
			HCQ	HCQ 0.8 g single dose	93	0	38/55	66 (16)	–	
			LPV/r	LPV/r 400 mg b.i.d.; For >75 years old, LPV/r 400 mg q.d.a.m. + LPV/r 200 mg q.d.p.m.	83	0	37/46	63 (17)	5	
			Standard of Care	–	506	0	284/222	71 (20)	–	
Panagopoulos *et al.* (2020)	Retrospective cohort	Greece	LPV/r + HCQ + AZM	–	8	–	2/6	56 (19)	–	[38]
			HCQ + AZM	–	8	–	4/4	60 (11)	–	
RECOVERY (2020)	Parallel RCT	United Kingdom	LPV/r	0.4 g LPV + 0.1 g RTV b.i.d.	1616	–	643/973	66 (16)	5	[32]
			Standard of Care	–	3424	–	1320/2104	66 (16)	–	
Chen *et al.* (2020)	Retrospective cohort	China	LPV/r + ARB	ARB, LPV/r 500 mg b.i.d	35	0	23/12	43 (31–48)	7	[98]
			ARB	ARB 200 mg b.i.d.	11	0	8/3	32 (29–65)	7	
Wen *et al.* (2020)	Retrospective cohort	China	LPV/r	0.2 g LPV + 0.05 g RTV x2 b.i.d.	59	0	32/27	52 (16)	7	[87]
			Standard of Care	Nursing, rest, symptom specific and supportive treatment, antibiotics	58	3	28/30	47 (16)	7	
			LPV/r + ARB	ARB, LPV/r	25	0	17/8	49 (17)	7	
			ARB	ARB 0.1 g x2 t.i.d.	36	0	20/16	53 (15)	7	
WHO Solidarity Trial (2020)	Parallel RCT	Multinational	LPV/r	0.2 g LPV + 0.05 g RTV x2 b.i.d.	1399	–	548/851		14	[33]
			Standard of care	–	1372	–	570/802	–	14	
Xu *et al.* (2020)	Retrospective cohort	China	LPV/r	0.2 g LPV + 0.05 g RTV x2 b.i.d.	64	8	40/24	51 (17)	5–10	[99]
			Standard of care	–	46	2	31/15	55 (18)	5–10	
Yan *et al.* (2020)	Retrospective cohort	China	LPV/r	0.4 g LPV + 0.1 g RTV b.i.d.	78	25	35/43	50 (34–61)	>10	[37]
			Standard of care	–	42	6	19/23	57 (37–66)		
Yao *et al.* (2020)	Retrospective cohort	China	LPV/r	0.2 g LPV + 0.05 g RTV x2 b.i.d.	19	6	13/6	51 (17)	7–10	[100]
			Standard of care	–	11	3	4/7	52 (12)	–	
Ye *et al.* (2020)	Retrospective cohort	China	LPV/r	0.4 g LPV + 0.1 g RTV b.i.d. or 0.8 g LPV + 0.2 g RTV q.d.	42	–	21/21	–	–	[36]
			Standard of care	–	5	–	4/1	–	–	
Yu *et al.* (2020)	Retrospective cohort	China	LPV/r	0.2 g LPV + 0.05 g RTV x2 b.i.d.	108	22	56/52	48 (16)	5	[101]
			Standard of care	–	114	13	61/53	51 (17)	5	

† Age is presented as mean (SD) or median (IQR) unless otherwise specified.

ARB: Arbidol/umifenovir; AZM: Azithromycin; F: Female; HCQ: Hydroxychloroquine; IFN: Interferon; LPV: Lopinavir; LPV/r: Lopinavir–ritonavir combination therapy; M: Male; RCT: Randomized controlled trial; RTV: Ritonavir.

### Risk of bias

**Figure 2.**
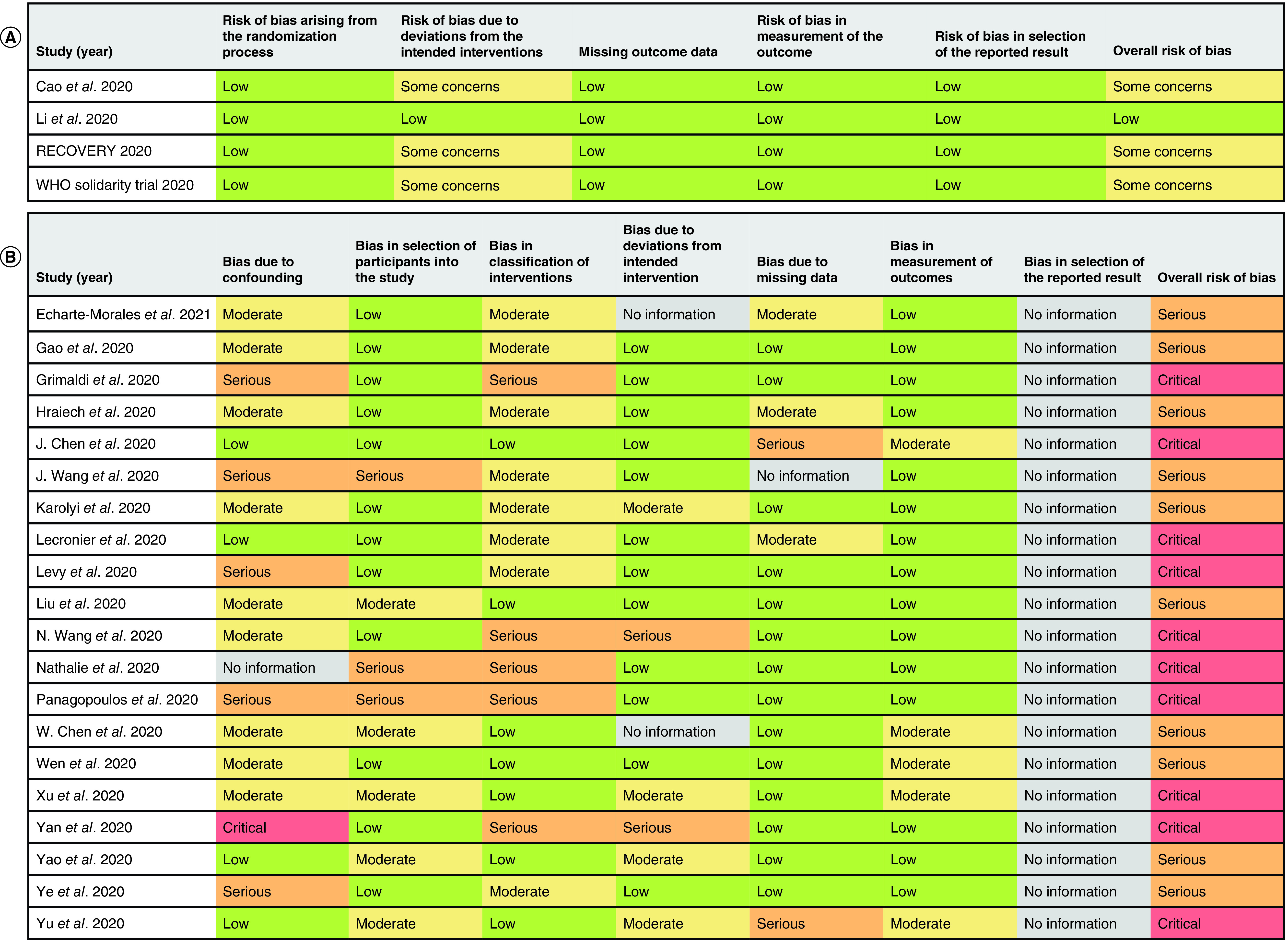
Risk of bias ratings for included studies. **(A)** Risk of bias ratings for randomized controlled trials using RoB2. **(B)** Risk of bias ratings for observational studies using ROBINS-I. RoB2: Revised Cochrane Risk of Bias Tool for Randomized Trial; ROBINS-I: Risk of bias in non-randomized study of intervention.

The risk of bias in the included RCTs was assessed using RoB2 (Figure 2A). Three RCTs [32–34] were rated as having some concerns for risk of bias due to their unblinded, open-label design. The remaining RCT by Li *et al.* [86] was rated as having a low risk of bias.

The risk of bias in included observational studies was assessed using ROBINS-I (Figure 2B). Ten studies [36,87,88,93–95,97,98,100,103] were rated as having a serious risk of bias, while ten studies [37,38,89–92,96,99,101,102] were rated as having a critical risk of bias. Five studies [36,38,89,90,103] were rated as having a serious risk of bias due to confounding factors, while one study [37] was rated as having a critical risk of bias due to either a lack of reporting for important patient characteristics (e.g., disease severity) or an imbalance in the reported patient characteristics. In terms of risk of bias due to selection of participants, three studies [38,92,103] were rated as having a serious risk of bias due to suspected selection bias, such as only including discharged patients in retrospective analyses. Five studies [37,38,90,92,96] were rated as having a serious risk of bias due to classification of interventions, mainly due to poorly defined inclusion criteria (e.g. studies that included patients who had received any LPV/r treatment during hospitalization without describing the LPV/r regimen). Two studies [37,96] were rated as having a serious risk of bias due to deviations from the intended interventions due to imbalances in the adjuvant therapy received between treatment arms. Lastly, two studies [101,102] were rated as having a serious risk of bias due to missing patient outcome data. Risk of bias due to selection of reported results cannot be evaluated for all observational studies because none of the studies provided their respective study protocols.

### Treatment efficacy

#### Mortality

**Figure 3.**
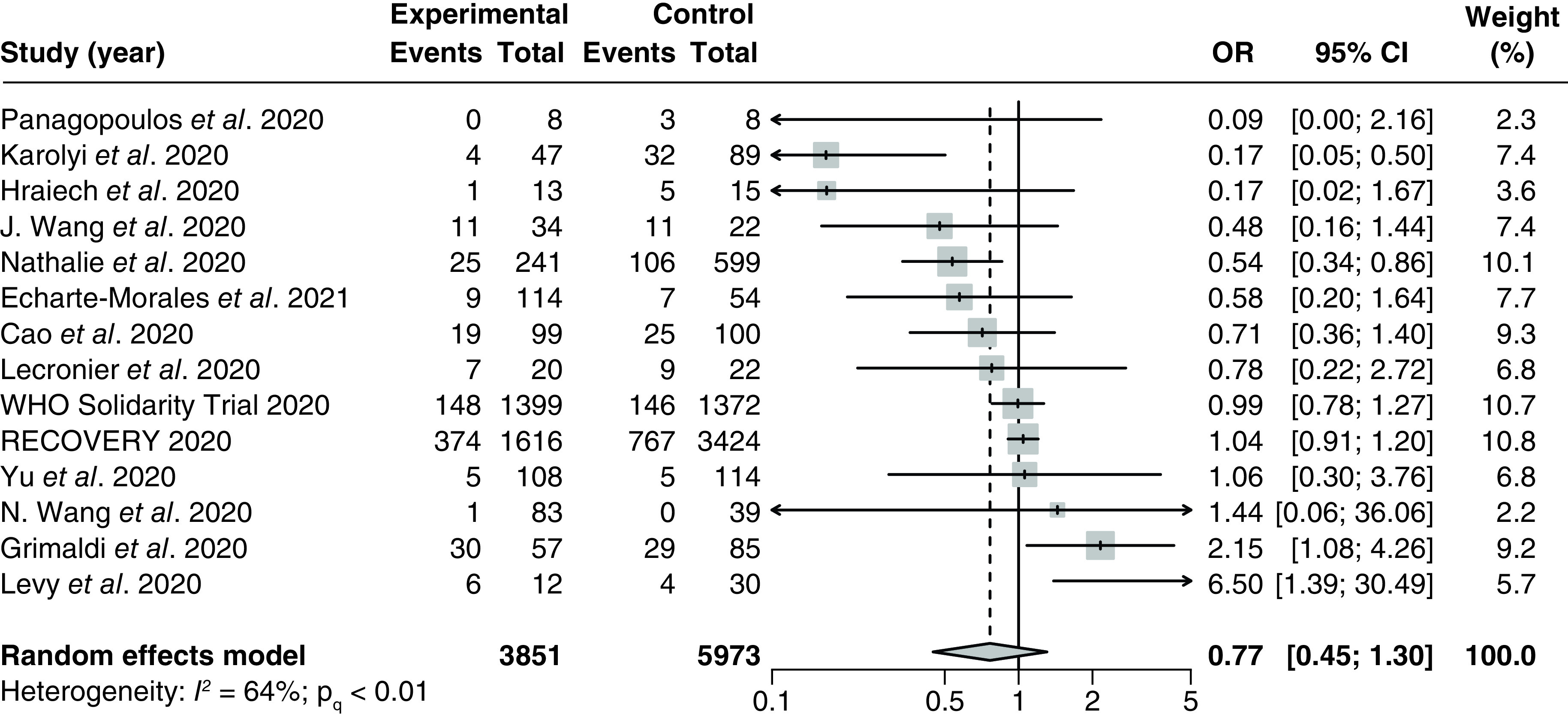
Forest plot for the pooling of odds ratios for mortality. The use of LPV/r was compared with control groups using standard of care or adjuvant therapies without LPV/r. Heterogeneity was quantified using I^2^ statistics. OR < 1 indicates beneficial treatment effects of LPV/r compared with the control groups. LPV/r: Lopinavir–ritonavir; OR: Odds ratio.

A total of 17 studies [32–34,37,38,86,88–96,101,103] (4 RCTs and 13 observational studies) with 10,105 patients examined the effect of LPV/r on mortality in hospitalized COVID-19 patients. Three studies [37,86,88] reported 0 death in all treatment arms and were excluded from the meta-analysis. The pooled OR was 0.77 (95% CI: 0.45–1.30) with significant and moderate heterogeneity (P_Q_ <0.01, I^2^ = 64%; Figure 3). The NNT was 26.2 patients.

There were no significant differences between the pooled ORs from any subgroups, although heterogeneity was substantially reduced for the pooled treatment effect from RCTs only (I^2^ = 0%; P_Q_ = 0.54; Supplementary Figure 1), studies utilizing a daily regimen of LPV/r 0.8 g (I^2^ = 25%; P_Q_ = 0.25; Supplementary Figure 2), and studies utilizing hydroxychloroquine as an adjuvant therapy (I^2^ = 0%; P_Q_ = 0.43; Supplementary Figure 3). There were no significant correlations between the proportion of patients with severe disease and the treatment effect based on the meta-regression analysis from six studies [34,91,92,96,101,103] (p = 0.69; Supplementary Figure 4). Additionally, there were also no significant correlations between the follow-up duration and the treatment effect based on meta-regression of six studies [32,34,91,93–95] (p = 0.20; Supplementary Figure 5).

There was no publication bias based on visual inspection of the funnel plot. This is corroborated by Egger's regression test, which did not detect any significant small study effect (P_Egger_ = 0.22; Supplementary Figure 6).

#### Length of stay

**Figure 4.**
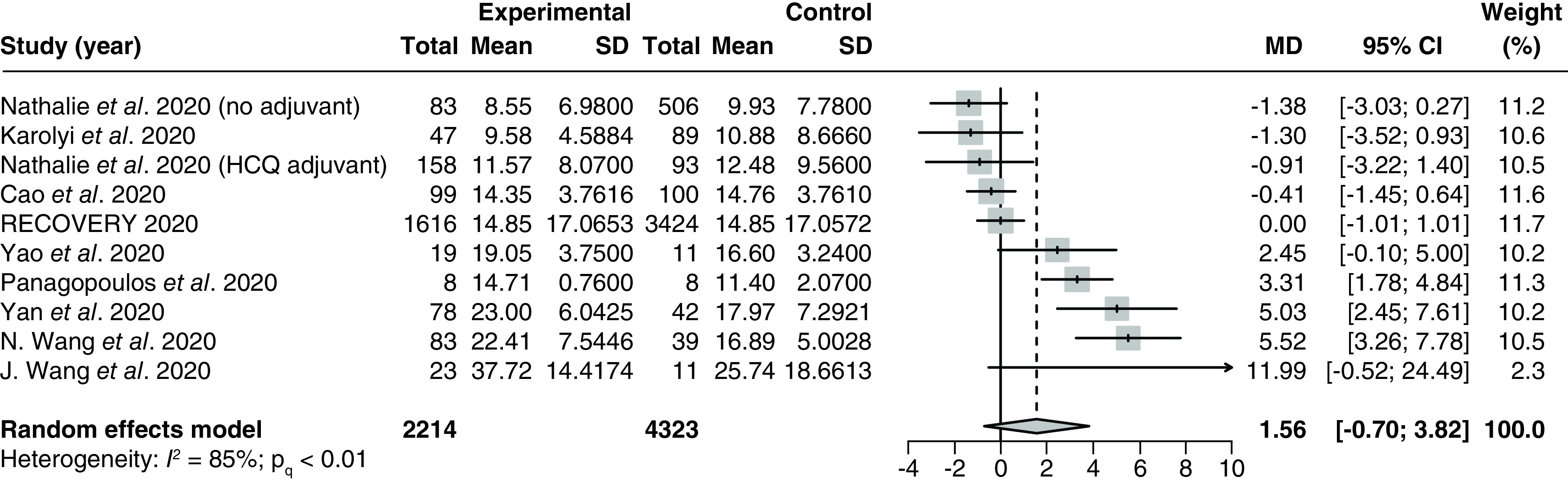
Forest plot for the pooling of mean differences for length of stay. The use of LPV/r was compared with control groups using standard of care or adjuvant therapies without LPV/r. Heterogeneity was quantified using I^2^ statistics. MD < 0 indicates beneficial treatment effects of LPV/r compared with the control groups. HCQ: Hydroxychloroquine; LPV/r: Lopinavir–ritonavir; MD: Mean difference.

A total of nine studies [32,34,37,38,92,94,96,100,103] (2 RCTs and 7 observational studies) with 6537 patients examined the effect of LPV/r on length of stay. An observational study conducted by Nathalie *et al.* [92] was included in the analysis as two separate entries, as the length of stay was reported separately for patients taking hydroxychloroquine adjuvants versus patients who were not taking additional adjuvants beyond standard of care. The pooled MD was 1.56 (95% CI: -0.70–3.82) with significant and severe heterogeneity (P_Q_ < 0.01; I^2^ = 85%; Figure 4).

There were significant between-group differences for the subgroup analysis by different adjuvant therapies, as a study conducted by Wang *et al.* [96] that used interferon (IFN) therapy as an adjuvant reported a considerably higher effect (MD: 5.52 [95% CI: 3.26–7.78]) compared with studies that used hydroxychloroquine adjuvants (MD: 1.30 [95% CI: -25.48–28.08]) and studies using no adjuvants (MD: 1.09 [95% CI: -2.01–4.20]). There were no significant differences in any of the other subgroup analyses (Supplementary Figures 7–9), although heterogeneity was significantly reduced in the RCT subgroup (P_Q_ = 0.58; I^2^ = 0%; Supplementary Figure 10).

Meta-regression analysis based on six studies [34,37,92,96,100,103] showed no significant correlations between the proportion of patients with severe disease and the treatment effect (p = 0.61; Supplementary Figure 11). There was no publication bias based on visual inspection of the funnel plot (Supplementary Figure 12).

#### Positive-to-negative conversion of SARS-CoV-2 nucleic acid test

**Figure 5.**
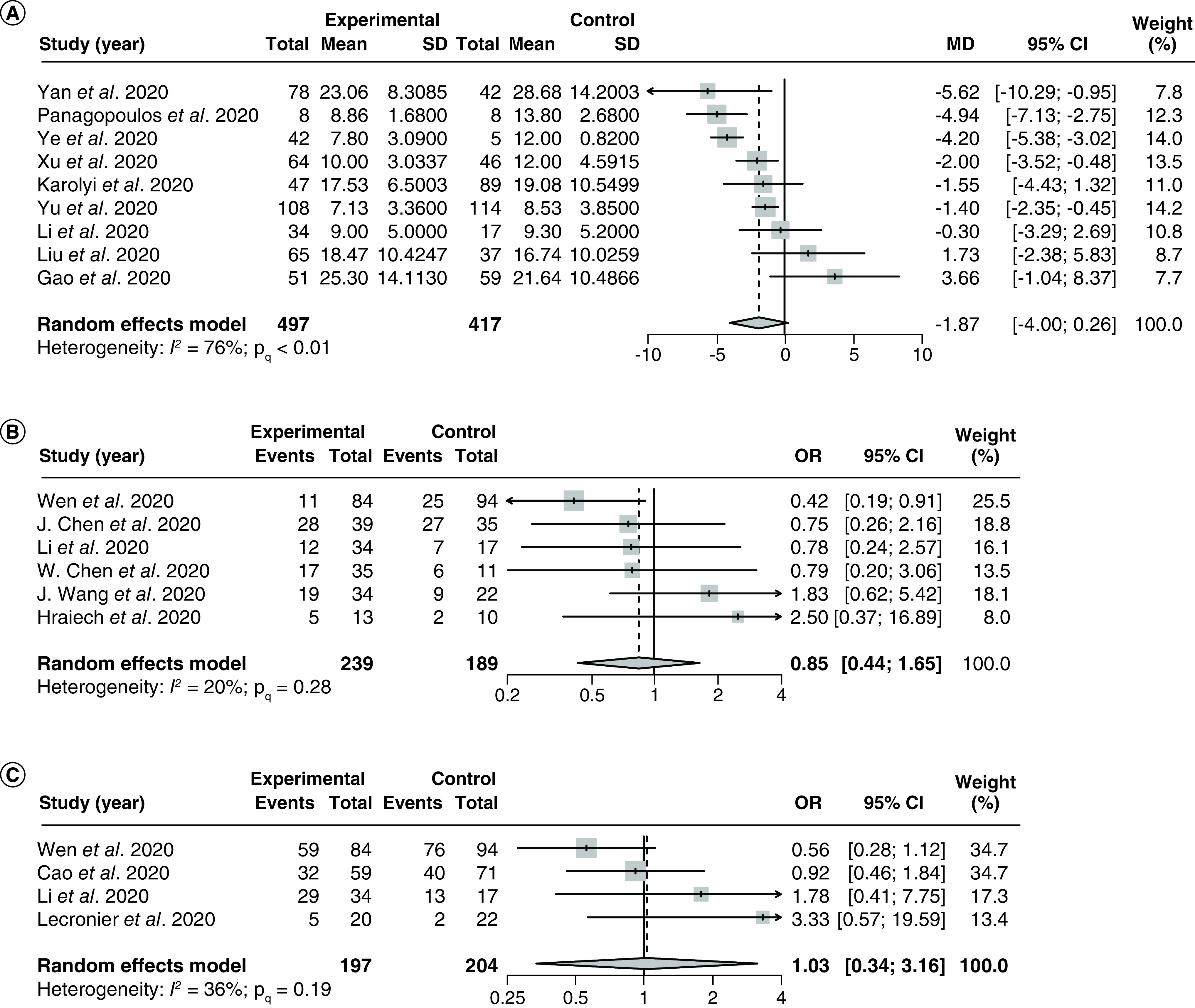
Forest plots for the pooling of mean differences for the outcome of time for positive-to-negative conversion of SARS-CoV-2 nucleic acid test and for the pooling of odds ratios for secondary efficacy outcomes. The use of LPV/r was compared with control groups using standard of care or adjuvant therapies without LPV/r. Heterogeneity was quantified using I^2^ statistics. **(A)** Forest plot for the pooling of MDs for the outcome of time for positive-to-negative conversion of SARS-CoV-2 nucleic acid test. MD < 0 indicates beneficial treatment effects of LPV/r compared with the control groups. **(B)** Forest plot for the pooling of ORs for incidences of positive-to-negative nucleic acid test conversions at day 7. **(C)** Forest plot for the pooling of ORs for incidences of positive-to-negative nucleic acid test conversions at day 14. OR >1 indicates beneficial treatment effects of LPV/r compared with the control groups for all secondary efficacy outcomes. LPV/r: Lopinavir–ritonavir; MD: Mean difference; OR: Odds ratio.

A total of nine studies [36–38,86,88,94,97,99,101] (1 RCTs and 8 observational studies) with 914 patients examined the effect of LPV/r on time for positive-to-negative conversion of SARS-CoV-2 nucleic acid tests. The pooled MD was -1.87 (95% CI: -4.00–0.26) with significant and severe heterogeneity (I^2^ = 76%; P_Q_ < 0.01; Figure 5A).

There were significant between-group differences for the subgroup analysis by different adjuvant therapies (Supplementary Figure 13), as a study conducted by Panagopoulos *et al.* [38] that used hydroxychloroquine and azithromycin as adjuvants reported a MD of -4.94 (95% CI: -7.13 to -2.75), which is substantially lower compared with the MD reported by Liu *et al.* [97] (MD: 1.73 [95% CI: -2.38–5.83]) which used IFN therapy as an adjuvant, as well as the pooled MD of studies that did not use adjuvants beyond standard of care (MD: -1.81 [95% CI: -4.16–0.55]). We did not perform the subgroup analysis by LPV/r regimens, as all studies included in the analysis reported using the same regimen of LPV/r 1.0 g q.d., with the exception of Panagopoulos *et al.* [38] which did not report the LPV/r regimens used in the study. There were no significant differences in the MDs between studies of different methodological designs, nor between studies that required imputation versus studies that did not require imputation (Supplementary Figures 14 & 15).

Meta-regression analysis based on 5 studies [37,86,88,99,101] showed no significant correlations between the proportion of patients with severe disease and the treatment effect (p = 0.10; Supplementary Figure 16). There was no publication bias based on visual inspection of the funnel plot (Supplementary Figure 17).

At 7 days after the commencement of LPV/r therapy, the OR of negative SARS-CoV-2 tests in the LPV/r group compared with the control group was 0.85 (95% CI: 0.44–1.65; NNH 25.4 patients) with no significant heterogeneity (I^2^ = 20%; P_Q_ = 0.28; Figure 5B) based on six studies [86,87,95,98,102,103]. At 14 days, the OR of negative SARS-CoV-2 tests was 1.03 (95% CI: 0.34–3.16, NNT 144.0 patients) with no significant heterogeneity (I^2^ = 36%; P_Q_ = 0.19, Figure 5C) based on four studies [34,86,87,91]. There were no significant differences between the pooled ORs from any subgroups for the aforementioned secondary outcomes (Supplementary Figures 18–22), nor were there any significant correlations between the proportion of patients with severe disease and the treatment effect based on meta-regression analyses (Supplementary Figures 23 & 24). There were no detectable small study effects in any of the aforementioned secondary outcomes based on visual inspections of the funnel plots (Figures 25 & 26).

#### Incidence of mechanical ventilation

**Figure 6.**
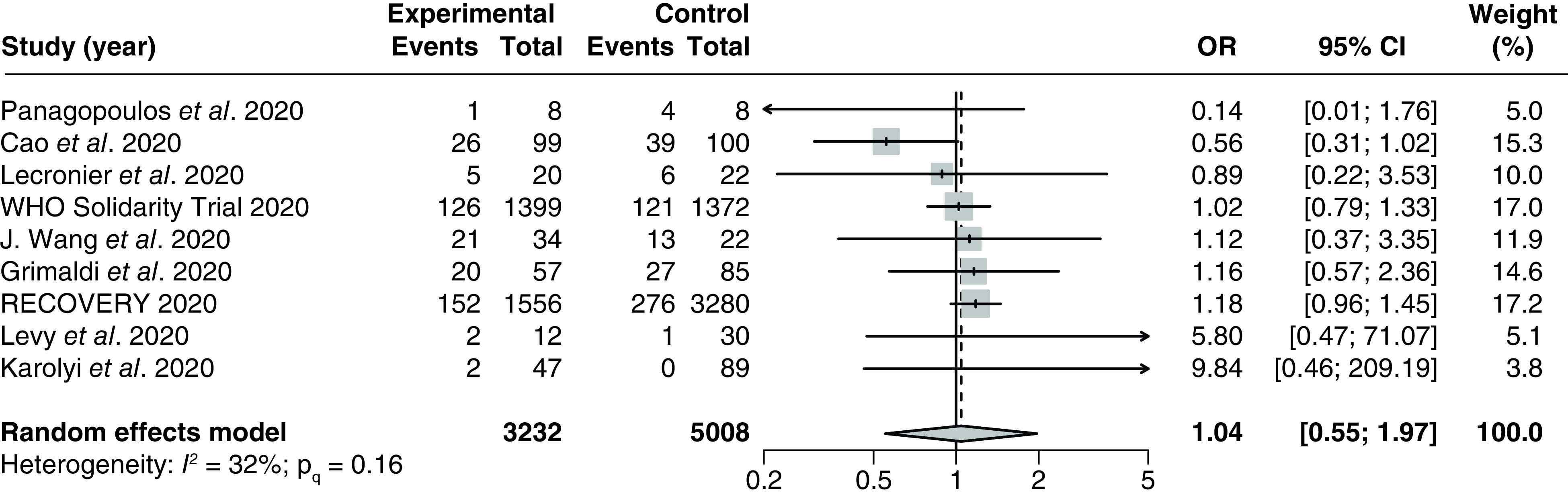
Forest plot for the pooling of odds ratios for incidence of mechanical ventilation. The use of LPV/r was compared with control groups using standard of care or adjuvant therapies without LPV/r. Heterogeneity was quantified using I^2^ statistics. OR < 1 indicates beneficial treatment effects of LPV/r compared with the control groups. LPV/r: Lopinavir–ritonavir; OR: Odds ratio.

Nine studies [17,33,34,38,89–91,94,103] (3 RCTs and 6 observational studies) with 8240 patients assessed the effect of LPV/r on incidences of mechanical ventilation in hospitalized COVID-19 patients. The pooled OR was 1.04 (95% CI: 0.55–1.97) with no significant heterogeneity (I^2^ = 32%; P_Q_ = 0.16; Figure 6). The NNH was 263.3 patients.

There were no significant between-group differences in any of the subgroup analyses (Supplementary Figures 27–29). We did not perform meta-regression analyses due to insufficient data. No evidence of small study effects was observed based on visual inspections of the funnel plot (Supplementary Figure 30).

#### Time to body temperature normalization

**Figure 7.**
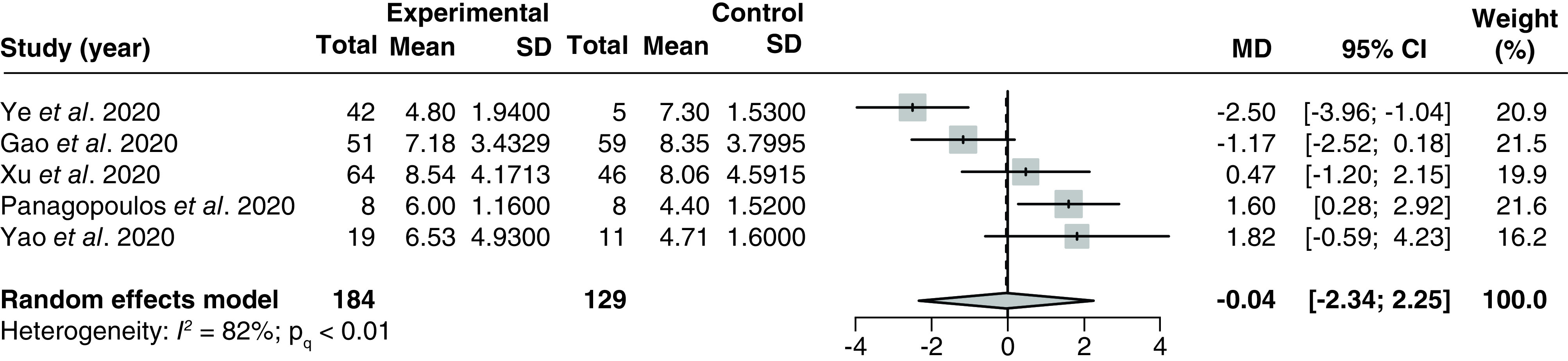
Forest plot for the pooling of mean differences for time to normalization of body temperature. The use of LPV/r was compared with control groups using standard of care or adjuvant therapies without LPV/r. Heterogeneity was quantified using I^2^ statistics. MD < 0 indicates beneficial treatment effects of LPV/r compared with the control groups. LPV/r: Lopinavir–ritonavir; MD: Mean difference.

Five studies [36,38,88,99,100] (all retrospective cohort studies) with 313 patients assessed the effect of LPV/r on time to body temperature normalization in hospitalized COVID-19 patients. The pooled MD was -0.04 (95% CI: -2.34–2.25) with significant and substantial heterogeneity (I^2^ = 82%; P_Q_ < 0.01; Figure 7).

There were no significant between-group differences in any of the subgroup analyses (Supplementary Figures 31–32). Meta-regression of outcomes from three studies [88,99,100] did not identify a significant correlation between the proportion of patients with severe disease and the treatment effect (p = 0.20; Supplementary Figure 33). We did not detect evidence of small study effects based on visual inspections of the funnel plot (Supplementary Figure 34).

#### Adverse events

**Figure 8.**
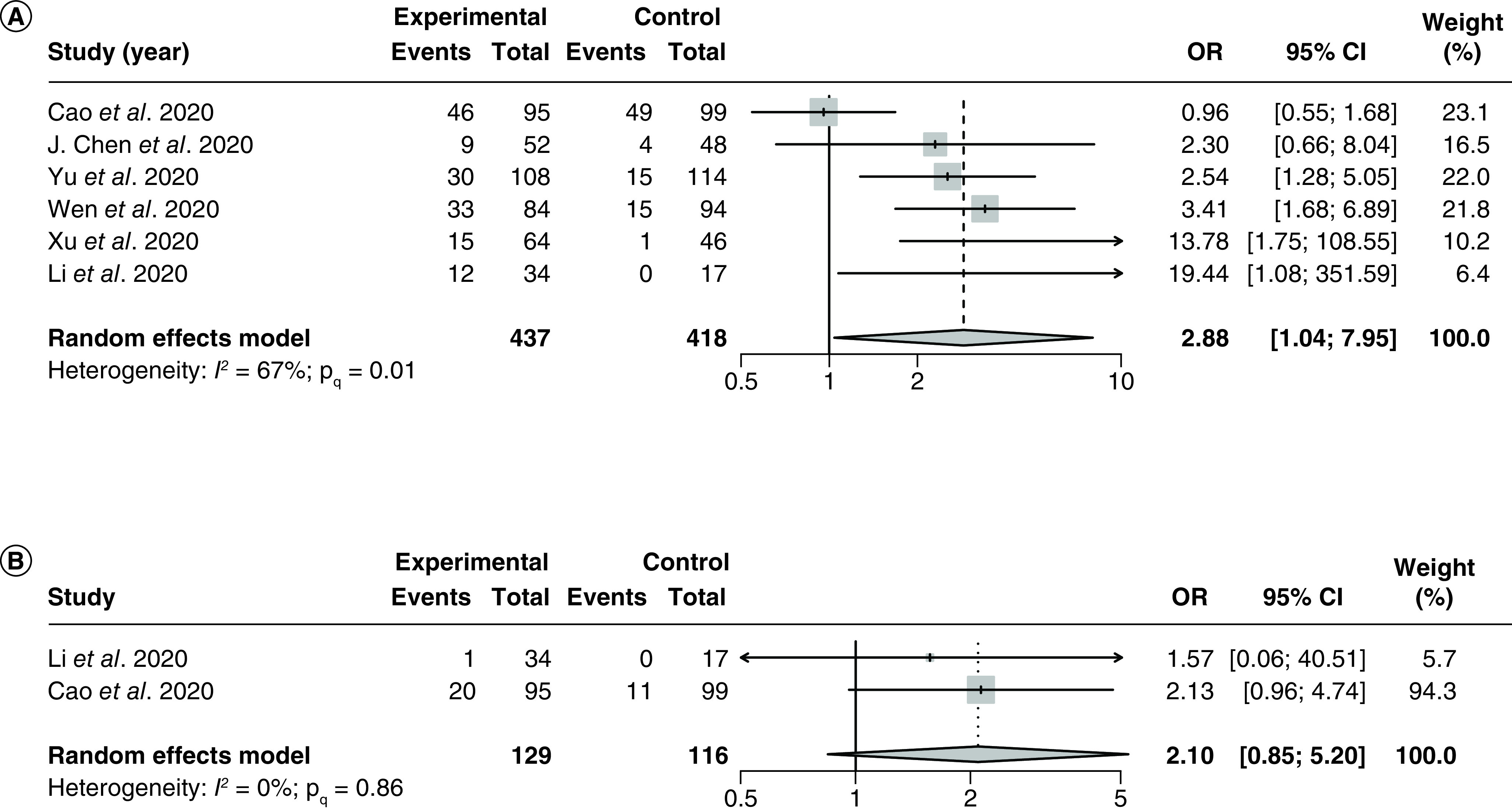
Forest plot for the pooling of odds ratios for incidence of adverse events and for pooling of odds ratios for secondary safety outcomes. The use of LPV/r was compared with control groups using standard of care or adjuvant therapies without LPV/r. Heterogeneity was quantified using I^2^ statistics. OR < 1 indicates better safety outcomes of LPV/r compared with the control groups for both forest plots. **(A)** Forest plot for the pooling of ORs for incidence of adverse events. **(B)** Forest plot for the pooling of ORs for incidence of severe adverse events. LPV/r: Lopinavir–ritonavir; OR: Odds ratio.

A total of six studies [34,86,87,99,101,102] (2 RCTs and 4 observational studies) with 855 patients examined the effect of LPV/r on incidences of adverse events in hospitalized COVID-19 patients. The pooled OR was 2.88 (95% CI: 1.04–7.95) with significant but moderate heterogeneity (I^2^ = 67%; P_Q_ < 0.01; Figure 8A). The NNH was 4.6 patients. The most commonly reported adverse events in the LPV/r group were gastrointestinal side effects such as diarrhea, nausea, vomiting, stomach pain and loss of appetite [34,87,89,99,101–103] and liver-related toxicities such as elevated liver enzymes and elevated bilirubin [89,101,103].

Subgroup analysis by different LPV/r regimens was not conducted as all studies included in the analysis reported using the same regimen of LPV/r 1.0 g q.d. There were no significant between-group differences in any of the subgroup analyses (Supplementary Figures 35 & 36). Meta-regression analysis of 6 studies [34,86,87,99,101,102] showed no significant correlations between the proportion of patients with severe disease and the treatment effect (p = 0.11; Supplementary Figure 37). Visual inspection of the funnel plot showed no significant small study effects (Supplementary Figure 38).

Two RCTs by Cao *et al.* [34] and Li *et al.* [86], which both used a regimen of LPV/r 1.0 g q.d. with no adjuvant therapy beyond standard of care, reported incidences of severe adverse events. The pooled OR was 2.10 (95% CI: 0.85–5.20), with no significant heterogeneity (I^2^ = 0%; P_Q_ = 0.86; Figure 8B). The NNH was 11.7 patients.

### Quality of evidence

The summary of findings for primary outcomes is tabulated in Table 2.

**Table 2.** Summary of findings, lopinavir–ritonavir therapy compared with standard of care/adjuvant therapies for the management of hospitalized COVID-19 patients.

Primary outcomes	Relative effect (95% CI)	Anticipated absolute effects (95% CI)^†^			Patients, n (studies, n)	Quality of evidence (GRADE)	Comments
		Risk without LPV/r	Risk with LPV/r	Risk difference (95% CI)			
Mortality	OR 0.77 (0.45–1.30)	189 per 1000	152 per 1000 (95 to 232)	37 fewer per 1000 (94 fewer to 43 more)	10,105 (4 RCTs, 13 OSs)	⊕◯◯◯ Very Low^‡^^,^^§^^,^^¶^	26 patients need to be treated with LPV/r to prevent one additional death.
Length of stay	–	The mean length of stay in the control groups was 14 days	–	MD 1.56 more days (0.70 fewer to 3.82 more)	6,537 (2 RCTs, 7 OSs)	⊕◯◯◯ Very Low^‡^^,^^§^^,^^¶^	
Time for positive-to-negative conversion of SARS-CoV-2 nucleic acid test	–	The mean time in the control groups was 16 days	–	MD 1.87 fewer days (4.00 fewer to 0.26 more)	914 (1 RCT, 8 OSs)	⊕◯◯◯ Very Low^‡^^,^^§^^,^^¶^	
Incidence of mechanical ventilation	OR 1.04 (0.55–1.97)	97 per 1000	100 per 1000 (56 to 175)	3 more per 1000 (41 fewer to 78 more)	8240 (3 RCTs and 6 OSs)	⊕⊕◯◯ Low^‡^^,^^¶^	263 patients need to be treated with LPV/r to cause one additional incidence of mechanical ventilation
Time to body temperature normalization	–	The mean time in the control groups was 8 days	–	MD 0.04 fewer days (2.34 fewer to 2.25 more)	313 (5 OSs)	⊕◯◯◯ Very Low^‡^^,^^§^^,^^¶^	
Incidence of adverse events	OR 2.88 (1.04–7.95)	201 per 1000	420 per 1000 (207 to 667)	219 more per 1000 (6 more to 466 more)	855 (2 RCTs and 4 OSs)	⊕⊕⊕◯ Moderate^‡^^,^^§^^,^^#^	5 patients need to be treated with LPV/r to cause one additional incidence of adverse events

† The risk in the intervention group (and its 95% Cl) is based on the assumed risk in the comparison group and the relative effect of the intervention (and its 95% CI).

‡ Downgraded due to study limitations; a majority of included studies were rated as having serious or critical risk of bias according to ROBINS-I.

§ Downgraded due to inconsistency; significant and severe heterogeneity was observed in the analysis.

¶ Downgraded due to imprecision; confidence intervals could not rule out the possibility of no effect (crosses null).

# Upgraded due to a large magnitude of effect.

GRADE Working Group quality of evidence rating [71].

High quality: We are very confident that the true effect lies close to that of the estimate of the effect.

Moderate quality: We are moderately confident in the effect estimate; the true effect is likely to be close to the estimate of the effect, but there is a possibility that it is substantially different.

Low quality: Our confidence in the effect estimate is limited; the true effect may be substantially different from the estimate of the effect.

Very low quality: We have very little confidence in the effect estimate; the true effect is likely to be substantially different from the estimate of effect.

GRADE: Grading of Recommendation, Assessment, Development and Evaluation; LPV/r: Lopinavir–ritonavir; MD: Mean difference; OR: Odds ratio; OS: Observational study; RCT: Randomized controlled trial.

## Discussions

### Main findings

Our systematic review and meta-analysis included 4 RCTs and 20 observational studies involving 10,718 hospitalized COVID-19 patients. On the basis of very low quality of evidence, we found that LPV/r use did not significantly decrease the odds of death, the length of stay, the time for positive-to-negative conversion of SARS-CoV-2 nucleic acid tests, or the time to normalization of body temperature. In fact, the use of LPV/r was associated with a non-significant increase in length of stay compared with standard of care or adjuvant therapies alone. We also did not find any benefits associated with LPV/r in reducing incidences of mechanical ventilation, based on a low quality of evidence.

The lack of efficacy in LPV/r use as shown by our primary outcomes is supported by results from our secondary outcomes as well, since we did not identify a significant increase in the incidence of positive-to-negative conversions of SARS-CoV-2 nucleic acid tests in the LPV/r group compared with the control group at 7 or 14 days after the start of treatment. In general, results of our subgroup analyses and meta-regressions did not yield any significant findings either, with the exception of two single-study subgroups: Wang *et al.* [96], which found that the use of an IFN adjuvant therapy with LPV/r significantly increased the length of stay compared with IFN therapy alone, and Panagopoulos *et al.* [38], which found that the use of hydroxychloroquine + azithromycin in combination with LPV/r resulted in significant reductions in the time for positive-to-negative conversion of SARS-CoV-2 nucleic acid tests compared with only hydroxychloroquine + azithromycin. However, as no data pooling was conducted for these subgroups, it is unknown whether these statistically significant results are due to subgroup factors or confounding variables unique to each study. Notably, Panagopoulos *et al.* [38] only included 16 patients in their study, which is the lowest sample size among all of our included publications. Previous investigations have found that studies with small sample sizes tend to overestimate treatment effects [104,105]; thus, the significant findings from Panagopoulos *et al.* [38] may be due to an exaggeration of the treatment effect away from the null and should be interpreted within the context of other similar studies. Nathalie *et al.* [92] and Echarte-Morales *et al.* [93] both used hydroxychloroquine as an adjuvant therapy with large sample sizes, and they did not find any significant benefits associated with using LPV/r in combination with hydroxychloroquine (with or without azithromycin) in other primary outcomes, including length of stay and death. This suggests that the results from single-study subgroups with low sample sizes should be interpreted with caution and require further examination in future investigations.

In addition to a lack of efficacy of LPV/r in the treatment of hospitalized COVID-19 patients, our meta-analysis also suggested that LPV/r may result in a significant 180% increase in the odds of adverse events based on moderate heterogeneity and moderate quality of evidence. While we did not specifically analyze the incidences of different categories of adverse events, gastrointestinal side effects and liver-related toxicities were the most commonly reported types of adverse events in the LPV/r group.

### Comparison to other studies

The lack of efficacy of LPV/r in COVID-19 patients is consistent with previous systematic reviews, which found that LPV/r did not significantly reduce time to virological cure, time to body temperature normalization, or incidences of cough relief [44]. LPV/r also did not decrease incidences of disease progression or mortality [45].

LPV/r was considered to be a potential treatment regimen for SARS-CoV-2 due to both *in vitro* and *in vivo* studies which demonstrated that it may be effective in inhibiting the action of other human coronaviruses, including SARS-CoV and MERS-CoV [21,23,106,107]. However, the most widely cited *in vivo* study conducted by Chu *et al.* [21] in SARS-CoV patients, which showed that LPV/r treatments may lower the severity of SARS-CoV symptoms, was an open, non-randomized trial involving a small treatment group and historical controls. The nature of the study design may be subjected to bias, and the results may be exaggerated due to improvements in disease management during the later stages of the 2004 epidemic.

In addition, many *in vitro* studies yielded different IC_50_ values in regards to the inhibitory effect of lopinavir on human coronaviruses, from 50 μmol/l in SARS-CoV [107] to 8–12 μmol/l in SARS-CoV-2 [108]. A study conducted by Wu *et al.* [107] attempted to synthesize lopinavir derivatives in an effort to improve the IC_50_ of lopinavir against SARS-CoV. However, they were only able to obtain a minimum IC_50_ of 25 μmol/l [107]. These values are generally greater than the maximum serum concentration (C_max_) of lopinavir, which has been estimated to be 13.5 μmol/l [109], and significantly greater than the unbound C_max_ of lopinavir, which has been estimated to be 0.2 μmol/l [110]. The IC_50_ values of lopinavir against coronaviruses are thousands of folds greater than the IC_50_ values required to inhibit HIV, which has been estimated to be around 0.004–0.011 μmol/l for lopinavir [108,111]. Given these findings, it has been suggested that the serum concentration of free LPV/r cannot suppress the action of SARS-CoV-2 *in vivo*, despite optimistic *in vitro* findings. This conclusion is supported by our current meta-analysis, which found that LPV/r lacks efficacy in a clinical setting.

Apart from the lack of efficacy, previous reviews have also identified an increased incidence of adverse events in the LPV/r group compared with standard of care [44], similar to our findings. Specifically, one review found that the use of LPV/r is significantly associated with a higher incidence of diarrhea [45], which we have also observed in five of our included studies [34,87,99,101,102] although we did not quantitatively analyze the incidence of diarrhea. In addition to gastrointestinal side effects, we also found hepatotoxicity to be widely reported across our included studies. Levy *et al.* [89] reported observing higher incidences of jaundice and bilirubin elevation in the LPV/r group compared with standard-of-care, Yu *et al.* [101] reported elevated transaminase in LPV/r patients, and J. Wang *et al.* [103] reported elevated aspartate aminotransferase and alanine aminotransferase. Gastrointestinal side effects and hepatotoxicity are both known adverse events associated with the use of LPV/r [112], with some studies reporting a prevalence of liver toxicities as high as 10% in HIV patients using LPV/r [113,114]. Levy *et al.* [89] noted that hepatotoxicity associated with LPV/r may be particularly problematic for the treatment of COVID-19 patients with obesity, as underlying liver steatosis may be a contributing factor to liver toxicity.

Recent reports have also raised concerns with regards to potential drug–drug interactions associated with LPV/r in the treatment of COVID-19 [115]. When used as a monotherapy, lopinavir has poor oral bioavailability because it is rapidly metabolized by cytochrome P450 enzyme systems [116]. For this reason, ritonavir is often co-formulated with lopinavir to take advantage of ritonavir's ability to potently inactivate cytochrome P4503A4 (CYP3A4), thus dramatically increasing the serum availability of lopinavir [117,118]. However, inhibition of CYP3A4 may also increase the serum concentration of other drugs that rely on CYP3A4 for metabolism, potentially resulting in drug–drug interactions [119]. For example, systemic corticosteroids (i.e. dexamethasone), which have been recently recommended for use in COVID-19 patients receiving supplemental oxygen [120,121], relies on CYP3A4 for metabolism [122]. Thus, the co-administration of corticosteroids and LPV/r may result in Cushing's syndrome and adrenal suppression [123,124]. Apart from COVID-19 treatments, medications prescribed for other conditions such as statins [125] and antiarrhythmic drugs [126] may also result in drug–drug interactions with LPV/r [115]. A previous observational study by Macías *et al.* [115] concluded that drug–drug interactions which contraindicates the use of LPV/r is often overlooked in the context of the ongoing COVID-19 healthcare crisis, signifying that physicians treating COVID-19 patients using LPV/r should be aware of potential interactions associated with CYP3A4 inhibition.

While the guidance from the NIH [127] and IDSA [128] currently recommend against the use of LPV/r, these are primarily based upon major RCTs, namingly the RECOVERY and SOLIDARITY trial. The use of LPV/r for the management of COVID-19 patients continues to be recommended in the clinical guidelines across several major countries, including China [40], Egypt, Saudi Arabia, Belgium and Ireland [41]. Our systematic review incorporated both randomized and observational evidence, and considered the impact of dosage on patient-important outcomes to provide comprehensive evidence on LPV/r's lack of efficacy. Additionally, given the significant number of adverse events associated with LPV/r, our findings could inform the perspectives of clinicians, policy makers and the general public to ensure patient safety.

### Strengths & limitations

This systematic review and meta-analysis has several strengths. First, we performed several subgroup analyses examining the impact of our study methodologies on the treatment effect, such as comparing the pooled effect from studies that required imputation of the mean and SD with studies that did not require imputation. Secondly, we examined the effect of different LPV/r regimens and adjuvant therapies on the treatment outcomes using subgroup analyses. And lastly, we compared the results from studies with a low risk of bias (which were all RCTs, coincidentally) with the results from studies with a high risk of bias (which were all observational and non-randomized studies). Compared with previous meta-analyses, our systematic review included more studies involving a larger sample size of patients, which helped improve the power and precision of our analyses. This was possible, in part, due to additional database searches conducted in Chinese literature sources. Additionally, we evaluated the quality of our evidence using the GRADE framework, which was not done in previous reviews.

However, our study also has several key limitations. Firstly, our subgroup analyses by different regimens and adjuvants often consist of many single-study subgroups, which severely limits the applicability of our subgroup analyses. Furthermore, a majority of our included studies are non-randomized observational studies, which are prone to biases and increased heterogeneity. According to ROBINS-I, all of our included observational studies were rated as having a serious or critical risk of bias, mainly due to potential confounding factors or inadequate descriptions of treatment regimes. Recent reports have shown that there has been a general decline in the quality of research articles and clinical trials [129,130] during the COVID-19 pandemic due to the increased pace of publications, which may explain the poorer quality of the articles that we included. Nevertheless, the pooled results from our observational studies were consistent with the pooled results from high or moderate quality RCT studies according to our subgroup analyses, which increases the confidence in our findings.

## Conclusion

This systematic review and meta-analysis indicates, based on low to very low quality of evidence, that the use of LPV/r is not significantly associated with reductions in mortality, length of stay, time for positive-to-negative conversion of SARS-CoV-2 nucleic acid test, incidence of mechanical ventilation, or time to body temperature normalization in hospitalized patients with COVID-19. We also found that the use of LPV/r is significantly associated with an increase in adverse events, based on moderate quality of evidence. Considering the lack of efficacy and the risk of increased gastrointestinal and liver-related toxicities, as well as potential drug–drug interactions with other COVID-19 treatments, the use of LPV/r therapy for treating COVID-19 inpatients is not recommended based on the available evidence.

**Summary points**

- Lopinavir–ritonavir (LPV/r) combination therapy is an antiretroviral medication that was repurposed for the treatment of COVID-19 due to promising results from *in vivo* and *in silico* studies involving human coronaviruses.
- In this systematic review and meta-analysis, results from 20 observational studies and 4 randomized controlled trials (n = 10,718) were included to examine the efficacy and safety of LPV/r for treating hospitalized COVID-19 patients.
- A majority of the included RCTs were rated as having some concerns in regards to risk of bias using RoB2, while all of the included observational studies were rated as having serious or critical risk of bias using ROBINS-I.
- The use of LPV/r was not associated with any significant benefit compared with standard of care or adjuvant therapies alone in reducing mortality, length of stay, time for positive-to-negative conversion of SARS-CoV-2 nucleic acid test, incidence of mechanical ventilation, or time to body temperature normalization.
- The use of LPV/r was significantly associated with increased odds of adverse events (OR: 2.88; 95% CI: 1.04–7.95). The most common adverse events observed in the LPV/r group included gastrointestinal side effects and possible hepatotoxicity.
- All efficacy outcomes were based on low to very low quality of evidence, while the outcome of adverse event incidence was based on moderate quality of evidence, according to the GRADE approach.
- Due to the lack of efficacy and increased odds of adverse events, the clinical use of LPV/r for the treatment of hospitalized patients with COVID-19 is not recommended based on the available evidence.
