# Supplementary material for: Efficacy of lopinavir–ritonavir combination therapy for the treatment of hospitalized COVID-19 patients: a meta-analysis
Source: Future Virol. 2022 Jan 31:10.2217/fvl-2021-0066. doi: 10.2217/fvl-2021-0066 (PMC8815807; doi:10.2217/fvl-2021-0066)
Supplement: Supplementary file 1 [file supplementary_materials.docx]

**Future Virology**

**Efficacy of lopinavir-ritonavir combination therapy for the treatment of hospitalized COVID-19 patients: A meta-analysis**

**Supplemental Materials**

**Table S1** PRISMA checklist

| **Section/topic** | **#** | **Checklist item** | **Reported on page #** |
| --- | --- | --- | --- |
| **TITLE** | | | |
| Title | 1 | Identify the report as a systematic review, meta-analysis, or both. | Title Page |
| **ABSTRACT** | | | |
| Structured summary | 2 | Provide a structured summary including, as applicable: background; objectives; data sources; study eligibility criteria, participants, and interventions; study appraisal and synthesis methods; results; limitations; conclusions and implications of key findings; systematic review registration number. | 1 |
| **INTRODUCTION** | | | |
| Rationale | 3 | Describe the rationale for the review in the context of what is already known. | 2 |
| Objectives | 4 | Provide an explicit statement of questions being addressed with reference to participants, interventions, comparisons, outcomes, and study design (PICOS). | 3 |
| **METHODS** | | | |
| Protocol and registration | 5 | Indicate if a review protocol exists, if and where it can be accessed (e.g., Web address), and, if available, provide registration information including registration number. | 3 |
| Eligibility criteria | 6 | Specify study characteristics (e.g., PICOS, length of follow-up) and report characteristics (e.g., years considered, language, publication status) used as criteria for eligibility, giving rationale. | 4 |
| Information sources | 7 | Describe all information sources (e.g., databases with dates of coverage, contact with study authors to identify additional studies) in the search and date last searched. | 3 |
| Search | 8 | Present full electronic search strategy for at least one database, including any limits used, such that it could be repeated. | Table S2-S9 |
| Study selection | 9 | State the process for selecting studies (i.e., screening, eligibility, included in systematic review, and, if applicable, included in the meta-analysis). | 4 |
| Data collection process | 10 | Describe method of data extraction from reports (e.g., piloted forms, independently, in duplicate) and any processes for obtaining and confirming data from investigators. | 4 |
| Data items | 11 | List and define all variables for which data were sought (e.g., PICOS, funding sources) and any assumptions and simplifications made. | 4 |
| Risk of bias in individual studies | 12 | Describe methods used for assessing risk of bias of individual studies (including specification of whether this was done at the study or outcome level), and how this information is to be used in any data synthesis. | 4 |
| Summary measures | 13 | State the principal summary measures (e.g., risk ratio, difference in means). | 5 |
| Synthesis of results | 14 | Describe the methods of handling data and combining results of studies, if done, including measures of consistency (e.g., I^2^) for each meta-analysis. | 5 |
| Risk of bias across studies | 15 | Specify any assessment of risk of bias that may affect the cumulative evidence (e.g., publication bias, selective reporting within studies). | 5 |
| Additional analyses | 16 | Describe methods of additional analyses (e.g., sensitivity or subgroup analyses, meta-regression), if done, indicating which were pre-specified. | 5 |
| **RESULTS** | | | |
| Study selection | 17 | Give numbers of studies screened, assessed for eligibility, and included in the review, with reasons for exclusions at each stage, ideally with a flow diagram. | 6, Figure 1 |
| Study characteristics | 18 | For each study, present characteristics for which data were extracted (e.g., study size, PICOS, follow-up period) and provide the citations. | Table 1 |
| Risk of bias within studies | 19 | Present data on risk of bias of each study and, if available, any outcome level assessment (see item 12). | Figure 2 |
| Results of individual studies | 20 | For all outcomes considered (benefits or harms), present, for each study: (a) simple summary data for each intervention group (b) effect estimates and confidence intervals, ideally with a forest plot. | Figure 3-8 |
| Synthesis of results | 21 | Present results of each meta-analysis done, including confidence intervals and measures of consistency. | Figure 3-8 |
| Risk of bias across studies | 22 | Present results of any assessment of risk of bias across studies (see Item 15). | 6-9 |
| Additional analysis | 23 | Give results of additional analyses, if done (e.g., sensitivity or subgroup analyses, meta-regression [see Item 16]). | 6-9 |
| **DISCUSSION** | | | |
| Summary of evidence | 24 | Summarize the main findings including the strength of evidence for each main outcome; consider their relevance to key groups (e.g., healthcare providers, users, and policy makers). | 9-10 |
| Limitations | 25 | Discuss limitations at study and outcome level (e.g., risk of bias), and at review-level (e.g., incomplete retrieval of identified research, reporting bias). | 11 |
| Conclusions | 26 | Provide a general interpretation of the results in the context of other evidence, and implications for future research. | 12 |
| **FUNDING** | | | |
| Funding | 27 | Describe sources of funding for the systematic review and other support (e.g., supply of data); role of funders for the systematic review. | Title Page |

**Table S2** MEDLINE search strategy

| **Line Number** | **Search Phrase** | **Results** |
| --- | --- | --- |
| 1 | ((("Corona virinae" or "corona virus" or Coronavirinae or coronavirus or COVID or nCoV or hCoV) adj4 ("19" or "2019" or novel or new)) or (("Corona virinae" or "corona virus" or Coronavirinae or coronavirus or COVID or nCoV or hCoV) and (wuhan or china or chinese or hubei)) or "COVID-19" or "Corona virinae19" or "Corona virinae2019" or "corona virus19" or "corona virus2019" or Coronavirinae19 or Coronavirinae2019 or coronavirus19 or coronavirus2019 or COVID19 or COVID2019 or nCOV19 or nCOV2019 or "SARS Corona virus 2" or "SARS Coronavirus 2" or "SARS-COV-2" or "Severe Acute Respiratory Syndrome Corona virus 2" or "Severe Acute Respiratory Syndrome Coronavirus 2" or sarscov*).ti,ab,hw,kw,mp. or (Severe Acute Respiratory Syndrome Coronavirus 2 or COVID-19 or COVID-19 drug treatment or COVID-19 serotherapy or COVID-19 diagnostic testing or COVID-19 vaccine or spike glycoprotein, COVID-19 virus).os,ps,rs,ox,px,rx,nm | 104,539 |
| 2 | (lopinavir or ritonavir or lopinavir-ritonavir or LPV or norvir or kaletra).ab,kw,ti. | 8,146 |
| 3 | exp Ritonavir/ or exp Lopinavir/ | 5,046 |
| 4 | 2 or 3 | 8,991 |
| 5 | 1 and 4 | 892 |
| 6 | Limit 5 to yr="2020-Current" | 887 |

**Table S3** EMBASE search strategy

| **Line Number** | **Search Phrase** | **Results** |
| --- | --- | --- |
| 1 | ((("Corona virinae" or "corona virus" or Coronavirinae or coronavirus or COVID or nCoV or hCoV) adj4 ("19" or "2019" or novel or new)) or (("Corona virinae" or "corona virus" or Coronavirinae or coronavirus or COVID or nCoV or hCoV) and (wuhan or china or chinese or hubei)) or "COVID-19" or "Corona virinae19" or "Corona virinae2019" or "corona virus19" or "corona virus2019" or Coronavirinae19 or Coronavirinae2019 or coronavirus19 or coronavirus2019 or COVID19 or COVID2019 or nCOV19 or nCOV2019 or "SARS Corona virus 2" or "SARS Coronavirus 2" or "SARS-COV-2" or "Severe Acute Respiratory Syndrome Corona virus 2" or "Severe Acute Respiratory Syndrome Coronavirus 2" or sarscov*).ti,ab,hw,kw,mp. | 102,845 |
| 2 | (lopinavir or ritonavir or lopinavir-ritonavir or LPV or norvir or kaletra).ab,kw,ti. | 12,247 |
| 3 | exp Ritonavir/ or exp Lopinavir/ | 22,576 |
| 4 | 2 or 3 | 27,438 |
| 5 | 1 and 4 | 1,402 |
| 6 | limit 5 to yr="2020-Current" | 1,374 |

**Table S4** PubMed search strategy

| **Line Number** | **Search Phrase** |
| --- | --- |
| 1 | ("COVID-19"[Supplementary Concept] OR "severe acute respiratory syndrome coronavirus 2"[Supplementary Concept]) OR "COVID-19"[Title/Abstract]) OR "COVID19"[Title/Abstract]) OR "COVID2019"[Title/Abstract]) OR "COVID-2019"[Title/Abstract]) OR "SARS-CoV-2"[Title/Abstract]) OR "SARSCoV2"[Title/Abstract]) OR "sars coronavirus 2"[Title/Abstract]) OR "2019-nCoV"[Title/Abstract]) OR "2019nCoV"[Title/Abstract]) OR "nCoV2019"[Title/Abstract]) OR "nCoV-2019"[Title/Abstract]) OR (("Wuhan"[Title/Abstract] OR "Hubei"[Title/Abstract]) AND "coronavirus*"[Title/Abstract]) AND ("lopinavir"[Title/Abstract] OR "ritonavir"[Title/Abstract] OR "lopinavir-ritonavir"[Title/Abstract] OR "LPV"[Title/Abstract] OR "norvir"[Title/Abstract] OR "kaletra"[Title/Abstract]) |
| **Search Date Range Restriction:** 2020-2021 | |

**Table S5** CNKI search strategy

| **Line Number** | **Search Phrase** |
| --- | --- |
| 1 | (SU=('疫情'+'新冠'+'冠状'+'武汉'+'COVID-19' + '2019-nCoV' + 'coronavirus' + '2019nCoV' + 'SARS-CoV-2'+'COVID19'+'COVID'+'SARS-CoV'+'SARS') OR TI=('疫情'+'新冠'+'冠状'+'武汉'+'COVID-19' + '2019-nCoV' + 'coronavirus' + '2019nCoV' + 'SARS-CoV-2'+'COVID19'+'COVID'+'SARS-CoV'+'SARS') OR KY=('疫情'+'新冠'+'冠状'+'武汉'+'COVID-19' + '2019-nCoV' + 'coronavirus' + '2019nCoV' + 'SARS-CoV-2'+'COVID19'+'COVID'+'SARS-CoV'+'SARS')) AND (SU=('洛匹那韦'+'利托那韦'+'克力芝') OR TI=('洛匹那韦'+'利托那韦'+'克力芝') OR KY=('洛匹那韦'+'利托那韦'+'克力芝')) |
| **Search Date Range Restriction:** 1/1/2020-2/10/2021 | |

**Table S6** Wanfang search strategy

| **Line Number** | **Search Phrase** |
| --- | --- |
| 1 | (主题:（"疫情"OR"新冠"OR"冠状"OR"武汉"OR"COVID-19" OR "2019-nCoV" OR "coronavirus" OR "2019nCoV" OR "SARS-CoV-2" OR "COVID19" OR "COVID" OR "SARS-CoV" OR "SARS") OR 题名或关键词:（"疫情"OR"新冠"OR"冠状"OR"武汉"OR"COVID-19" OR "2019-nCoV" OR "coronavirus" OR "2019nCoV" OR "SARS-CoV-2" OR "COVID19" OR "COVID" OR "SARS-CoV" OR "SARS"））AND (主题:（"洛匹那韦" OR "利托那韦" OR "克力芝"）OR 题名或关键词:（"洛匹那韦" OR "利托那韦" OR "克力芝"）） |
| **Search Date Range Restriction:** 1/1/2020-2/10/2021 | |

**Table S7** Wanfang Med Online search strategy

| **Line Number** | **Search Phrase** |
| --- | --- |
| 1 | (TI=(疫情 OR 新冠 OR 冠状 OR 武汉 OR COVID-19 OR 2019-nCoV OR coronavirus OR 2019nCoV OR SARS-CoV-2 OR COVID19 OR COVID OR SARS-CoV OR SARS) OR KW=(疫情 OR 新冠 OR 冠状 OR 武汉 OR COVID-19 OR 2019-nCoV OR coronavirus OR 2019nCoV OR SARS-CoV-2 OR COVID19 OR COVID OR SARS-CoV OR SARS)) AND (TI=(洛匹那韦 OR 利托那韦 OR 克力芝) OR KW=(洛匹那韦 OR 利托那韦 OR 克力芝)) |
| **Search Date Range Restriction:** 1/1/2020-2/10/2021 | |

**Table S8** SinoMed search strategy

| **Line Number** | **Search Phrase** |
| --- | --- |
| 1 | ("疫情"[标题:智能] OR "新冠"[标题:智能] OR "冠状"[标题:智能] OR "武汉"[标题:智能] OR "COVID-19"[标题:智能] OR "2019-nCoV"[标题:智能] OR "coronavirus"[标题:智能] OR "2019nCoV"[标题:智能] OR "SARS-CoV-2"[标题:智能] OR "COVID19"[标题:智能] OR "COVID"[标题:智能] OR "SARS-CoV"[标题:智能] OR "SARS"[标题:智能]) AND ("洛匹那韦"[常用字段:智能] OR "利托那韦"[常用字段:智能] OR "克力芝"[常用字段:智能]) |
| **Search Date Range Restriction:** 1/1/2020-2/10/2021 | |

**Table S9** CQVIP search strategy

| **Line Number** | **Search Phrase** |
| --- | --- |
| 1 | (M=疫情 OR 新冠 OR 冠状 OR 武汉 OR COVID-19 OR 2019-nCoV OR coronavirus OR 2019nCoV OR SARS-CoV-2 OR COVID19 OR COVID OR SARS-CoV OR SARS) AND (M=糖皮质 OR 糖皮质素 OR 糖皮质激素 OR 激素 OR 肾上腺 OR 肾上腺激素 OR prednisone OR prednisolone OR 泼尼松 OR 泼尼松龙 OR dexamethasone OR 地塞米松 OR 地噻米松 OR 类固醇 OR 氟美松 OR 德沙美松 OR 氟甲强的松龙 OR 肾上腺素 OR 强的松 OR methylprednisolone OR 甲泼尼龙 OR 甲基泼尼松龙 OR 甲基强的松龙 OR 甲泼尼松龙 OR 甲强龙 OR 去氢可的松) AND (R=随机 OR 对照 OR 单盲 OR 双盲 OR 三盲 OR 开放) |
| **Search Date Range Restriction:** 1/1/2020-2/10/2021 | |

| **Figure S1** Forest plot for subgroup analysis by study design, mortality  There is no significant difference between the pooled odds ratio from randomized studies versus non-randomized studies (P=0.31). | **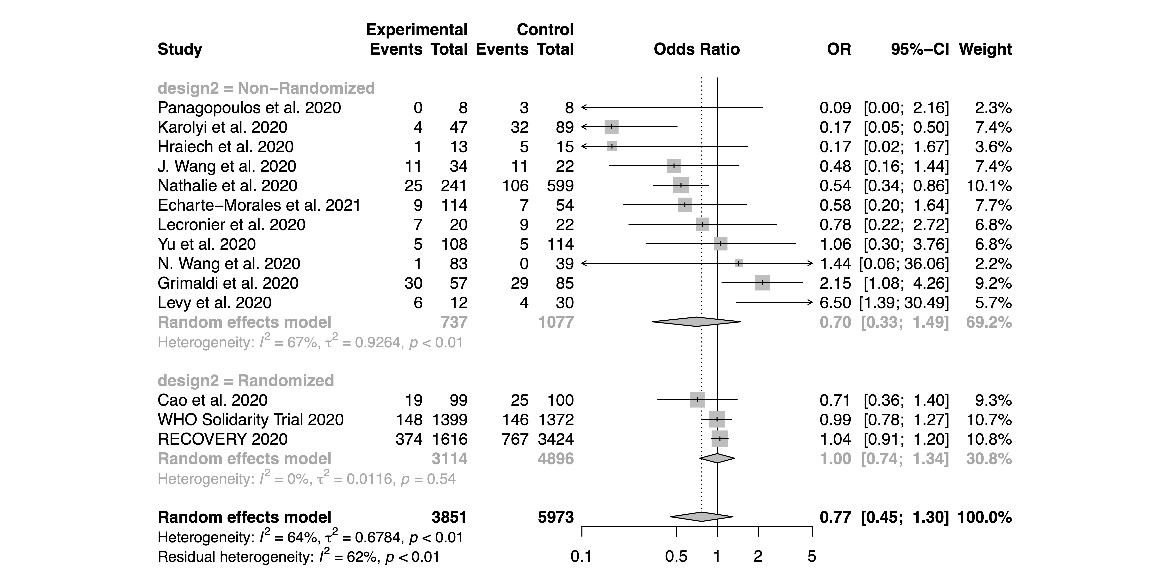** |
| --- | --- |
| **Figure S2** Forest plot for subgroup analysis by lopinavir/ritonavir regimens, mortality  There is no significant difference between the pooled odds ratio of different regimen subgroups (P=0.77). | **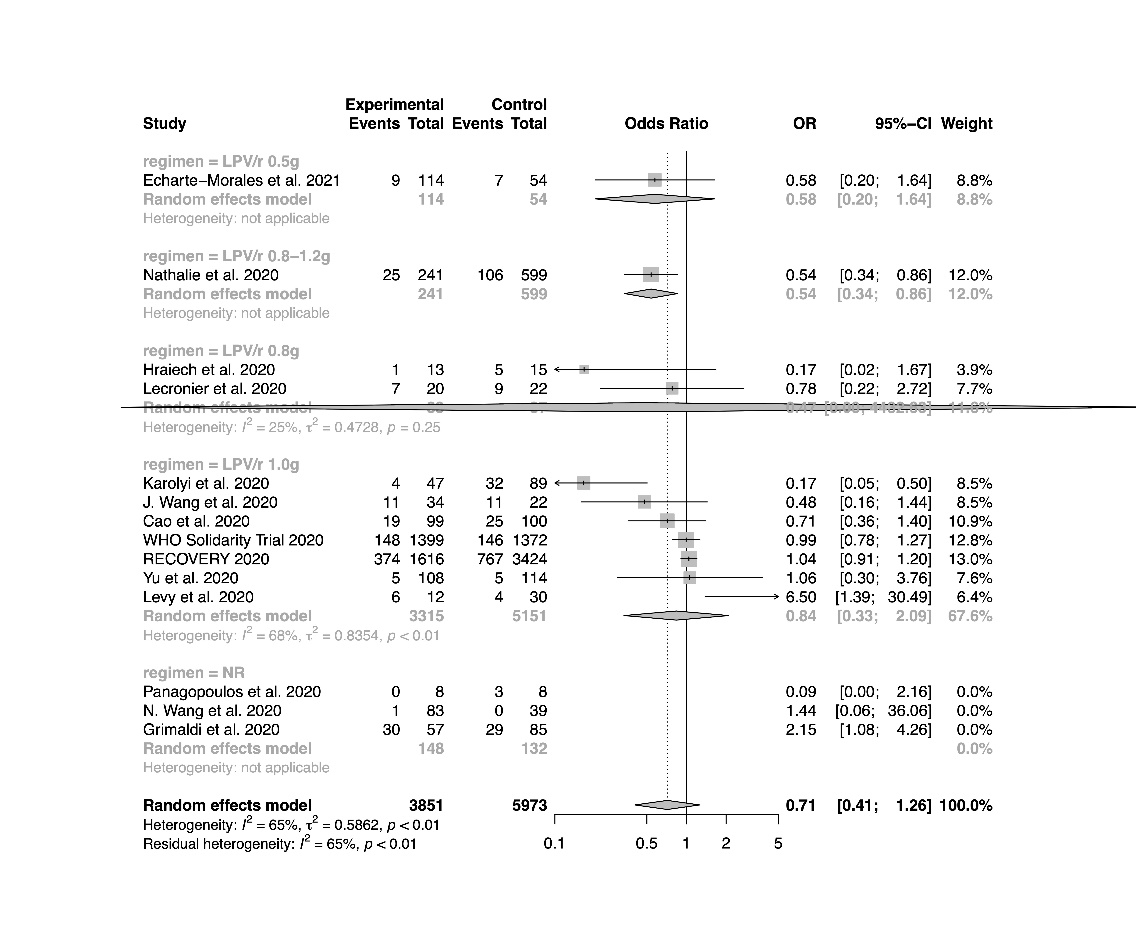** |
| **Figure S3** Forest plot for subgroup analysis by adjuvant therapies, mortality  There is no significant difference between the pooled odds ratio of different adjuvant subgroups (P=0.68). | 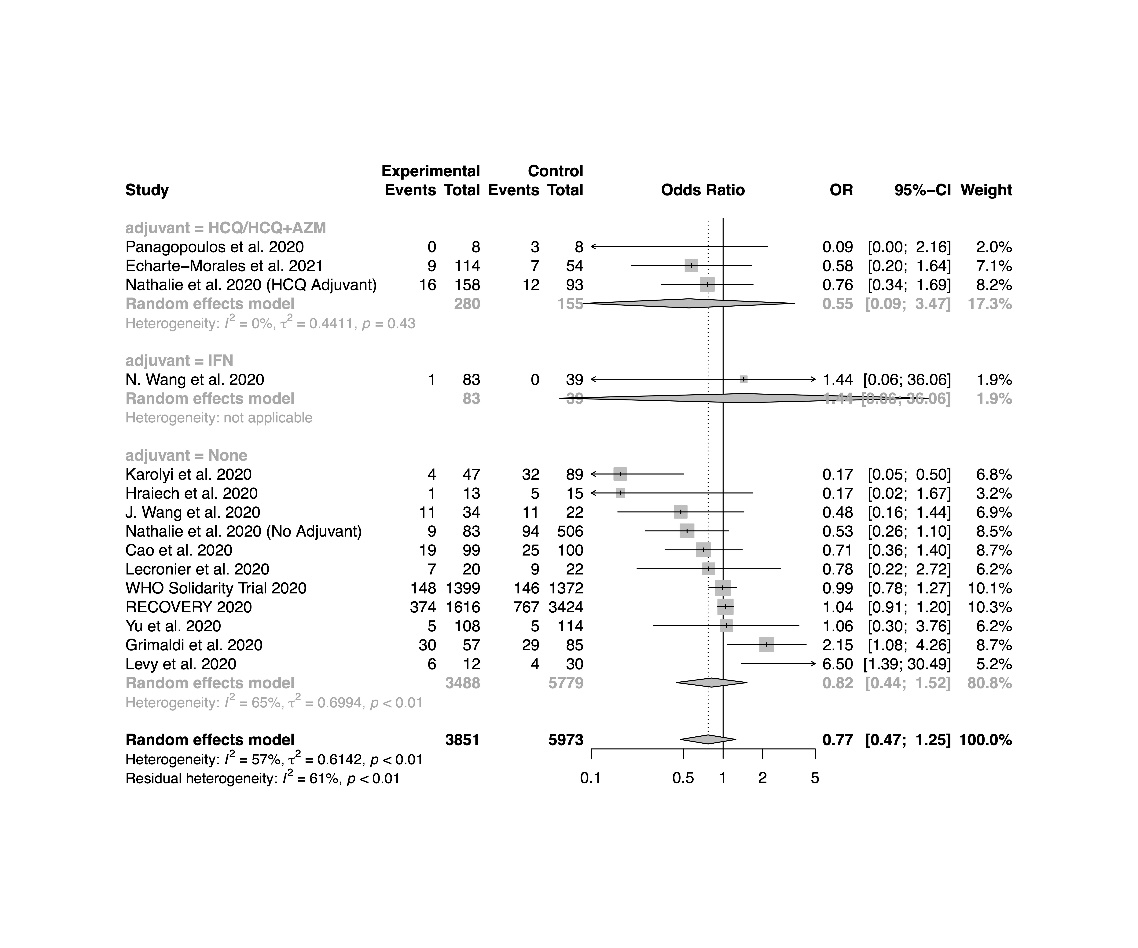 |
| **Figure S4** Bubble plot for meta-regression of proportion of patients with severe disease, mortality  There is no significant correlation between the proportion of patients with severe disease and the treatment effect (P=0.69). | **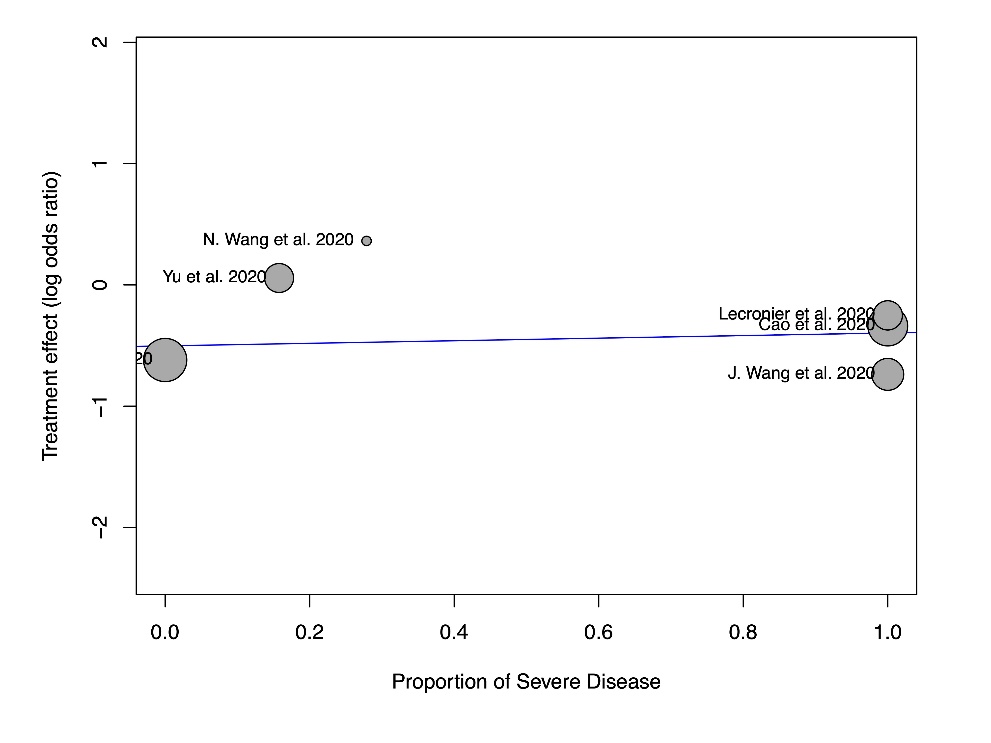** |
| **Figure S5** Bubble plot for meta-regression of follow up duration, mortality  There is no significant correlation between the follow up duration and the treatment effect (P=0.20). | **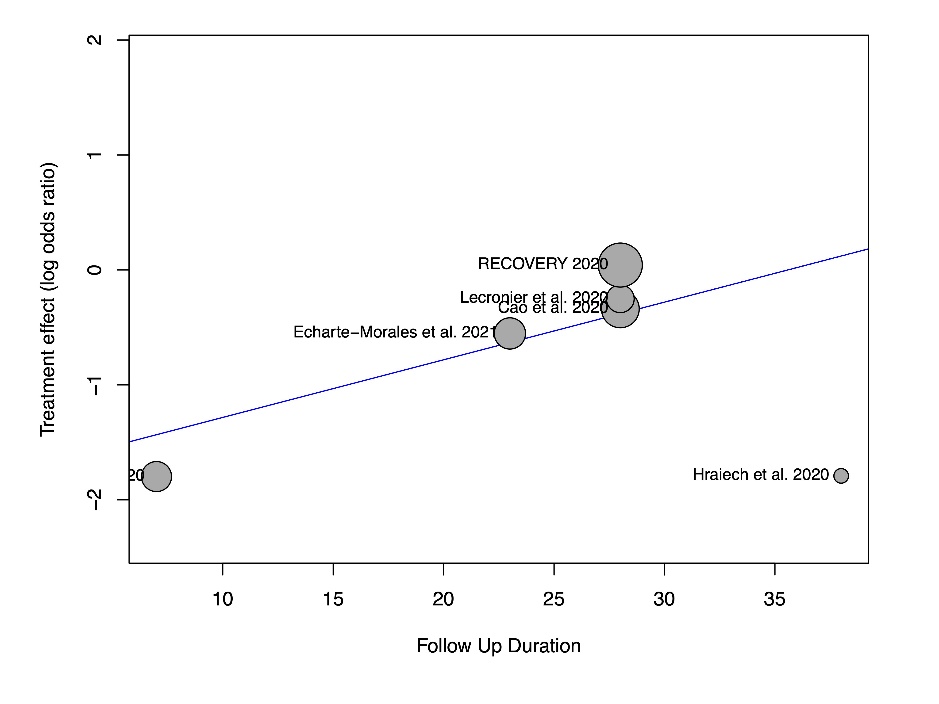** |
| **Figure S6** Funnel plot, mortality  There is no evidence of small study effects (P_Egger_=0.22). | **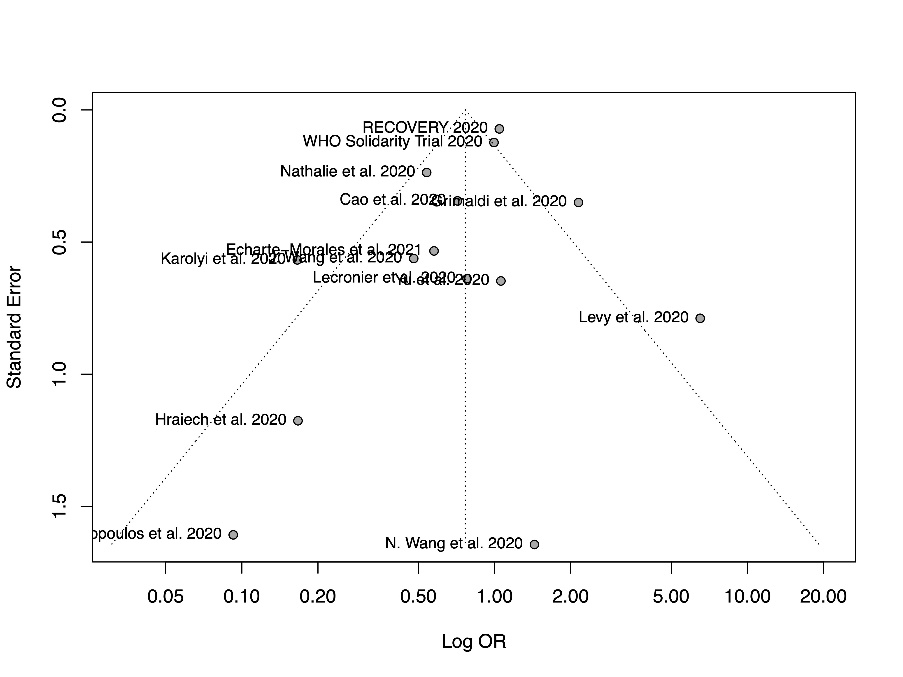** |
| **Figure S7** Forest plot for subgroup analysis by adjuvant therapies, length of stay  There are significant between-group differences between different adjuvant subgroups (P=0.02). | **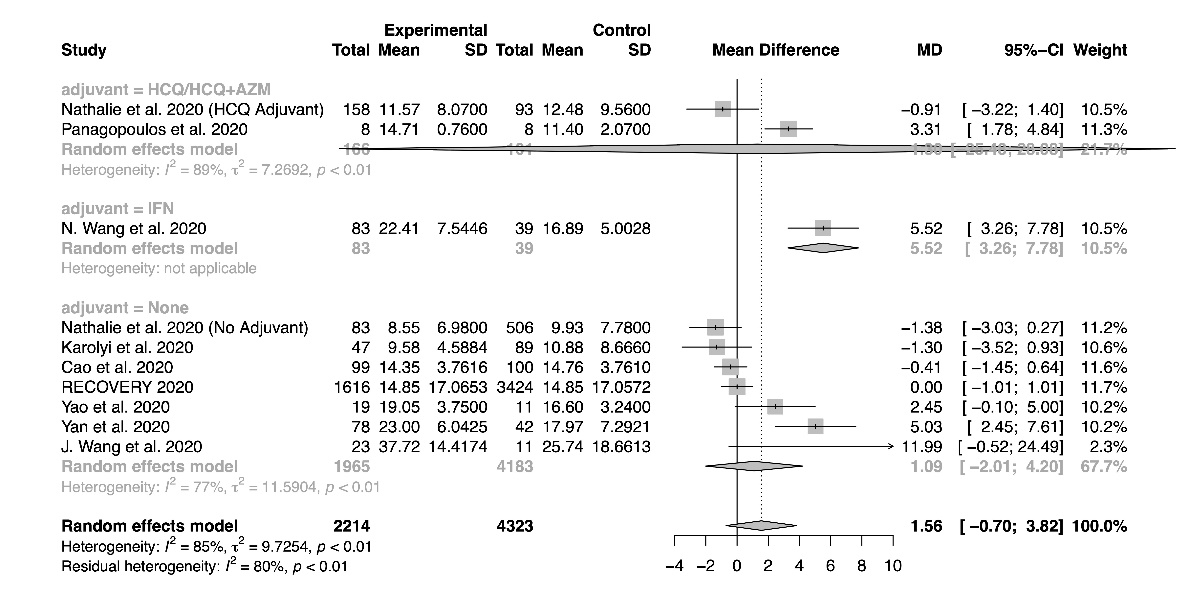** |
| **Figure S8** Forest plot for subgroup analysis by lopinavir/ritonavir regimens, length of stay  There is no significant difference between the pooled mean differences of different regimen subgroups (P=0.05). | **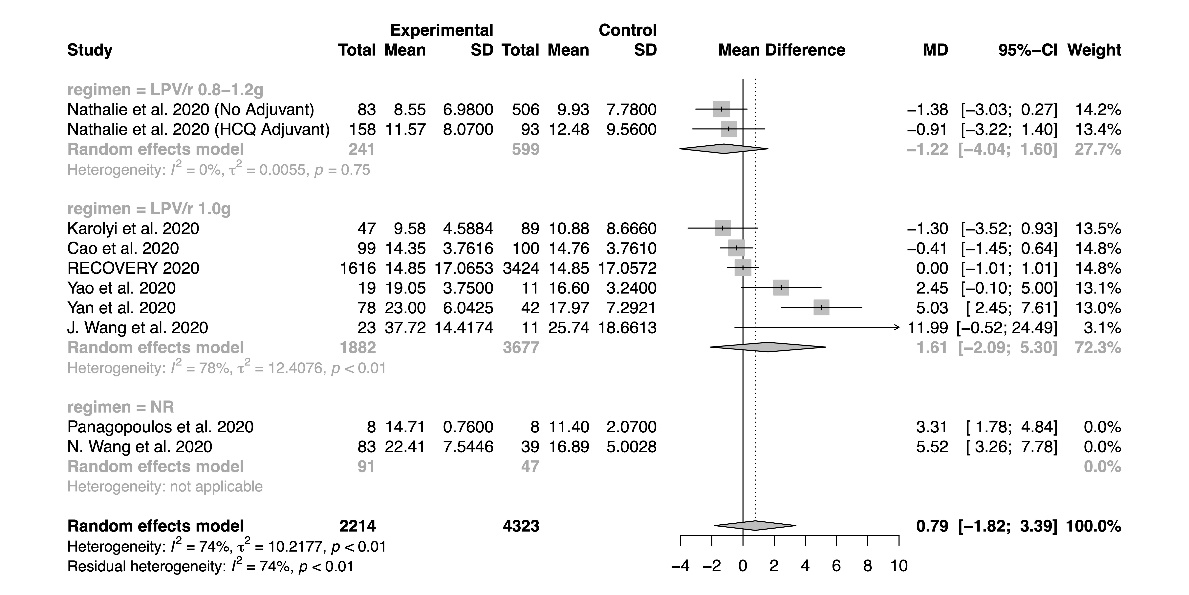** |
| **Figure S9** Forest plot for subgroup analysis by imputation, length of stay  There is no significant difference between the pooled mean differences of studies using imputation versus studies that do not require imputation (P=0.49). | **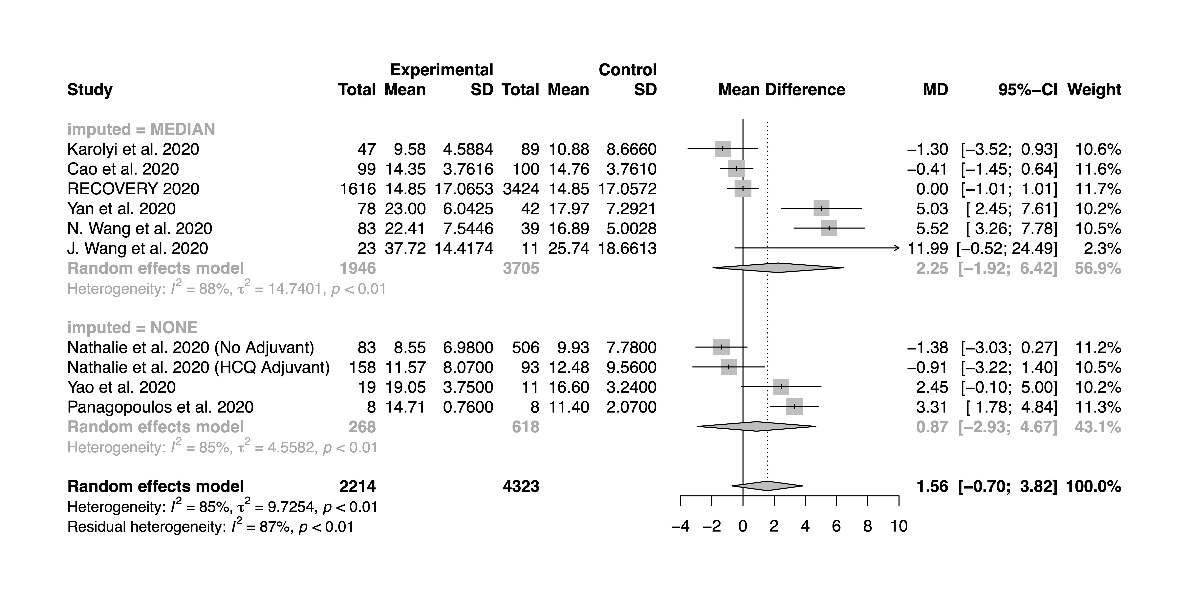** |
| **Figure S10** Forest plot for subgroup analysis by study design, length of stay  There is no significant difference between the pooled mean differences from randomized studies versus non-randomized studies (P=0.06). | **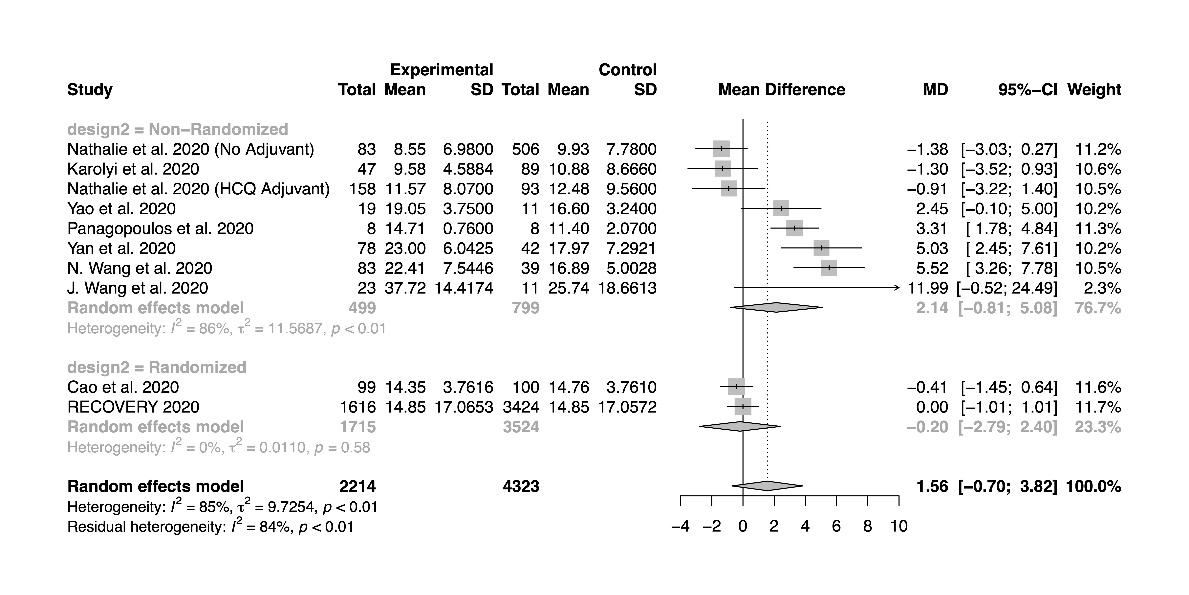** |
| **Figure S11** Bubble plot for meta-regression of proportion of patients with severe disease, length of stay  There is no significant correlation between the proportion of patients with severe disease and the treatment effect (P=0.61). | **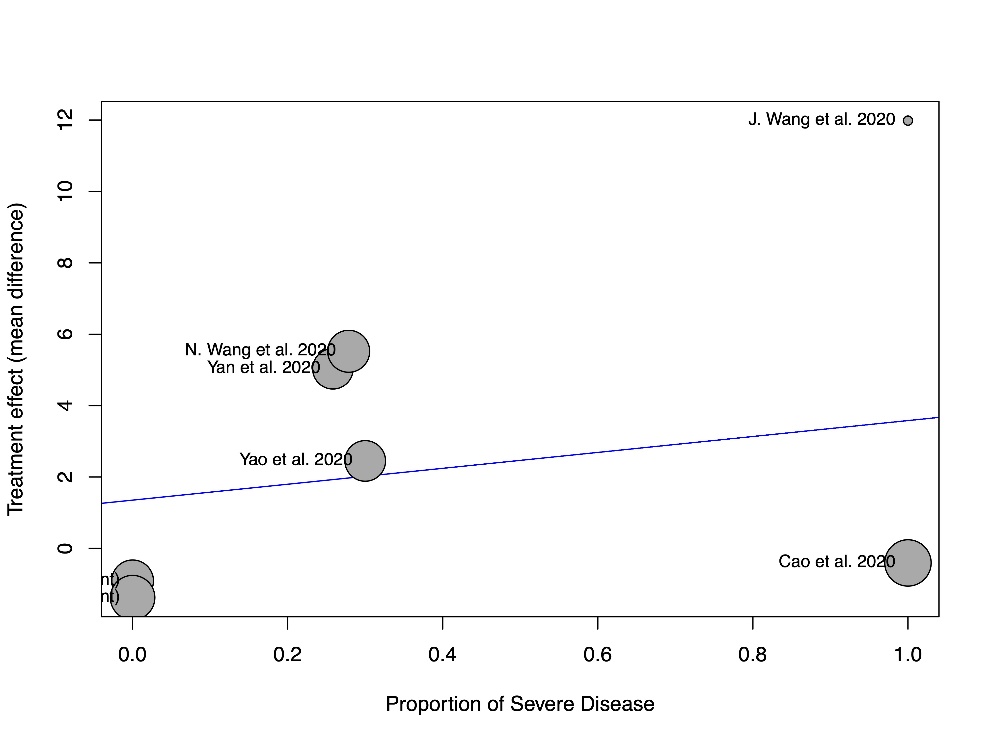** |
| **Figure S12** Funnel plot, length of stay  There is no evidence of small study effects based on visual inspection of the funnel plot. | **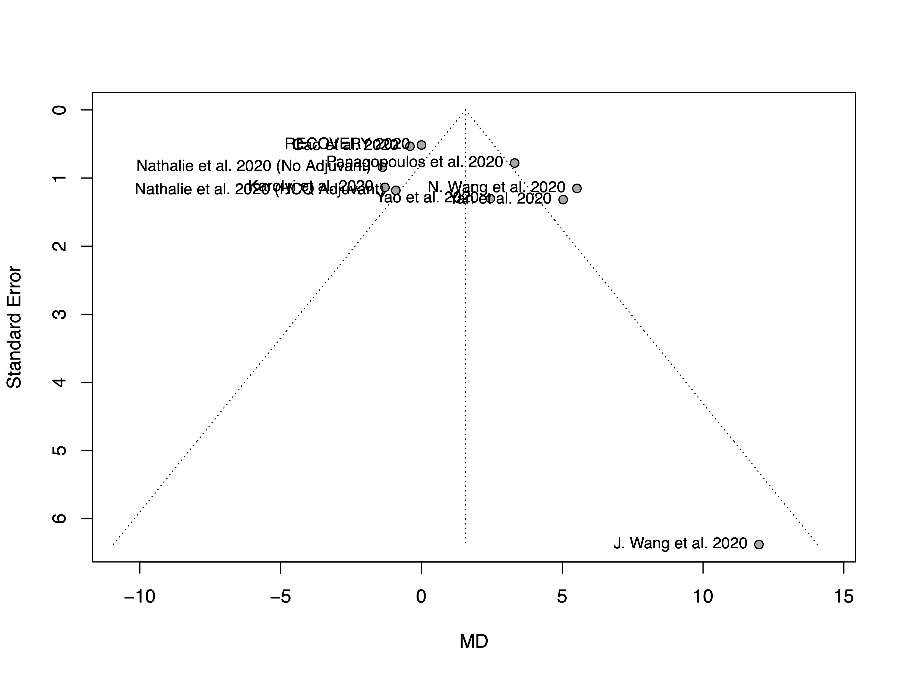** |
| **Figure S13** Forest plot for subgroup analysis by adjuvant therapies, time for positive-to-negative conversion of SARS-CoV-2 nucleic acid test  There are significant between-group differences between different adjuvant subgroups (P=0.01). | **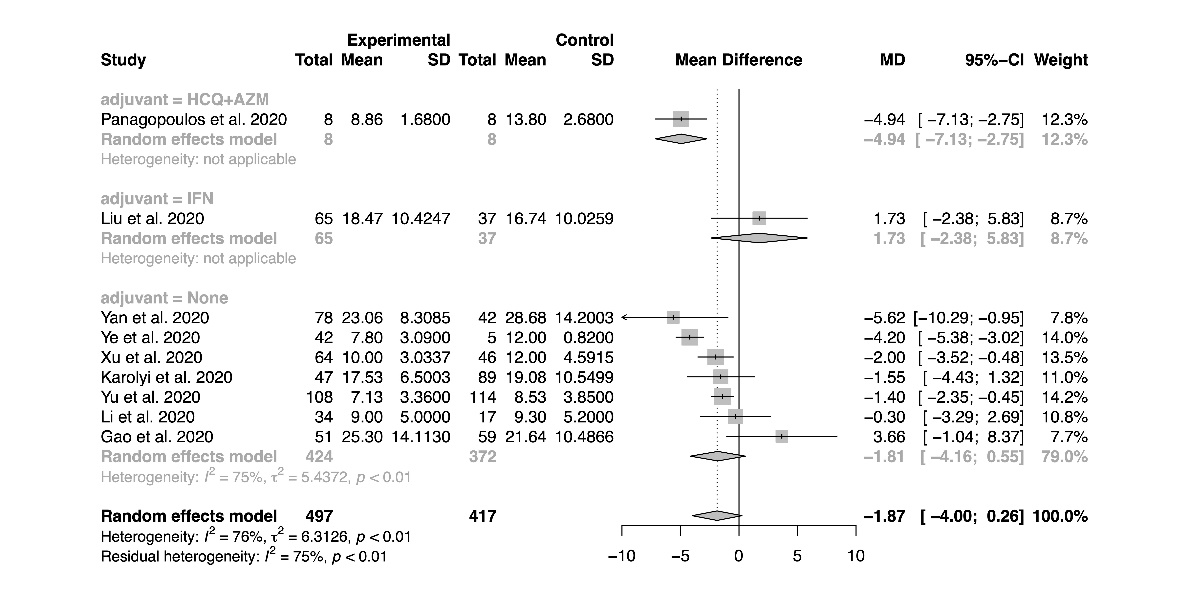** |
| **Figure S14** Forest plot for subgroup analysis by study design, time for positive-to-negative conversion of SARS-CoV-2 nucleic acid test  There is no significant difference between the pooled mean differences from randomized studies versus non-randomized studies (P=0.34). | **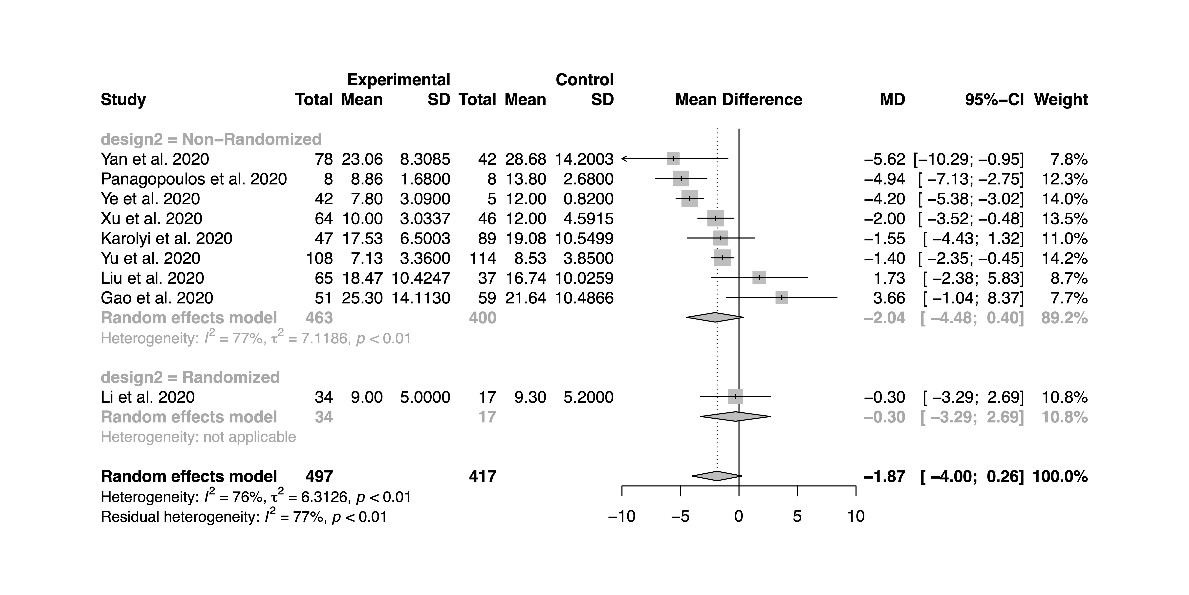** |
| **Figure S15** Forest plot for subgroup analysis by imputation, time for positive-to-negative conversion of SARS-CoV-2 nucleic acid test  There is no significant difference between the pooled mean differences of studies using imputation versus studies that do not require imputation (P=0.29). | **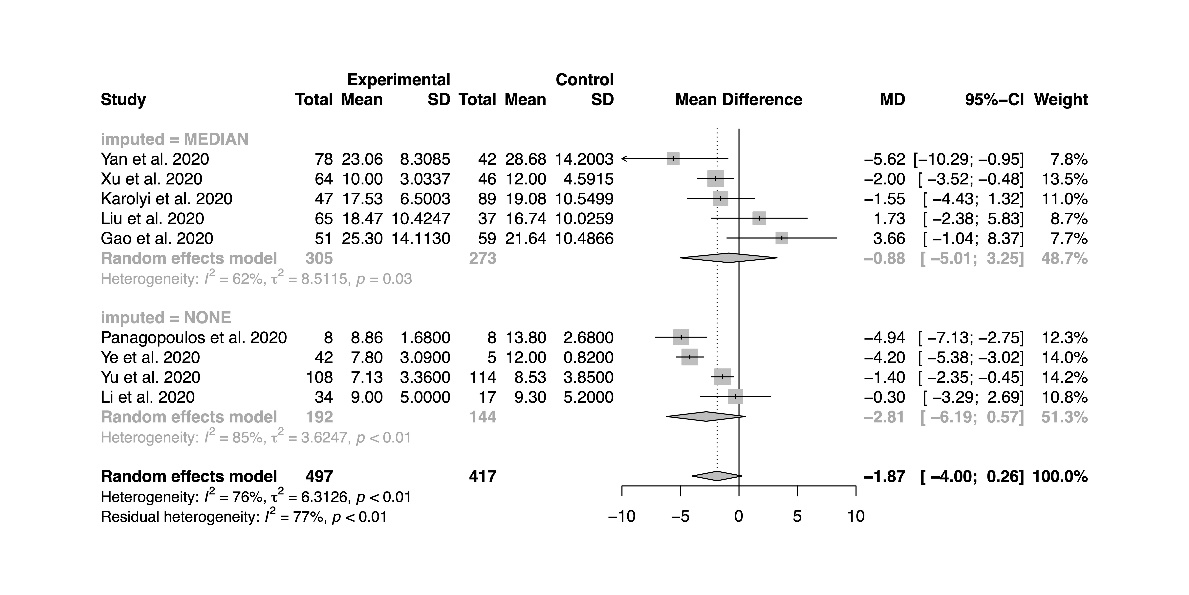** |
| **Figure S16** Bubble plot for meta-regression of proportion of patients with severe disease, time for positive-to-negative conversion of SARS-CoV-2 nucleic acid test  There is no significant correlation between the proportion of patients with severe disease and the treatment effect (P=0.10). | **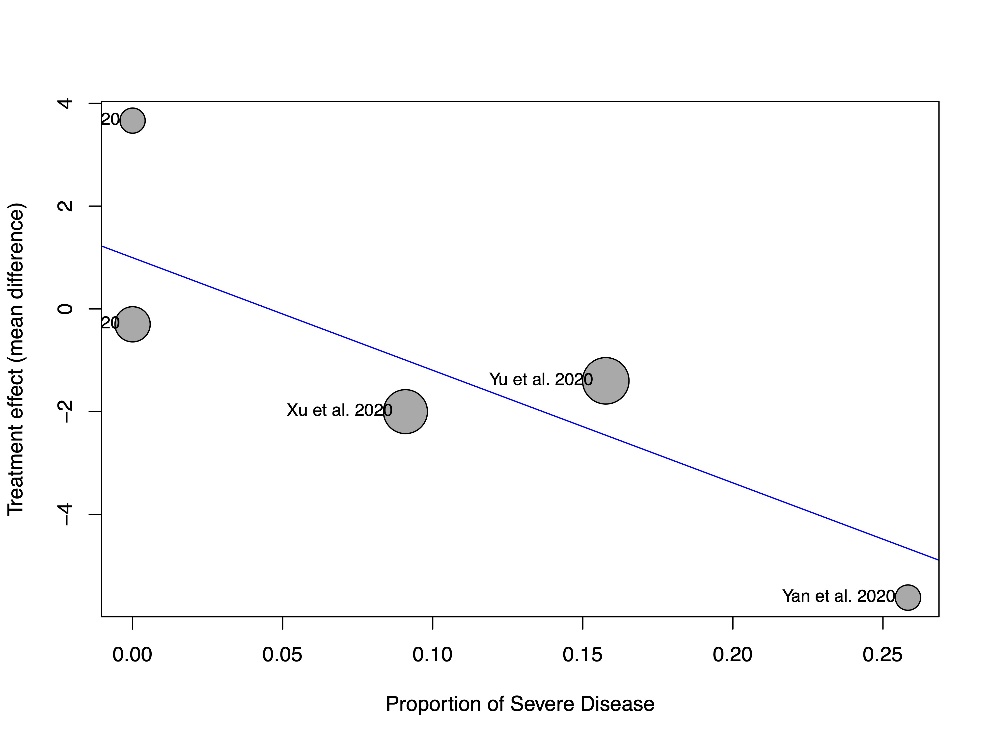** |
| **Figure S17** Funnel plot, time for positive-to-negative conversion of SARS-CoV-2 nucleic acid test  There is no evidence of small study effects based on visual inspection of the funnel plot. | **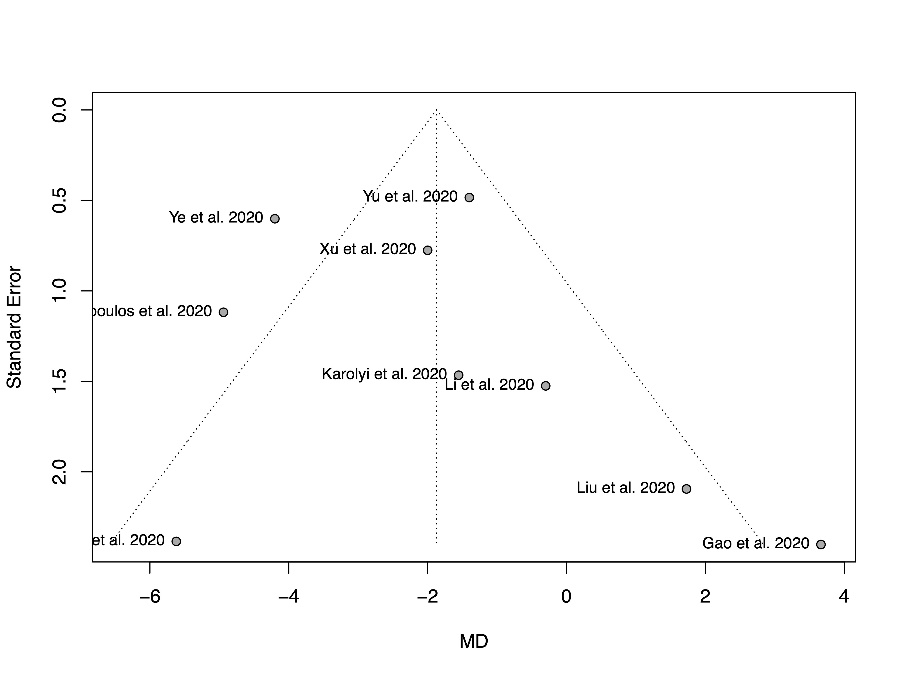** |
| **Figure S18** Forest plot for subgroup analysis by adjuvant therapies, incidence of positive-to-negative conversion of SARS-CoV-2 nucleic acid test at day 7  There are significant between-group differences between different adjuvant subgroups (P=0.98). | **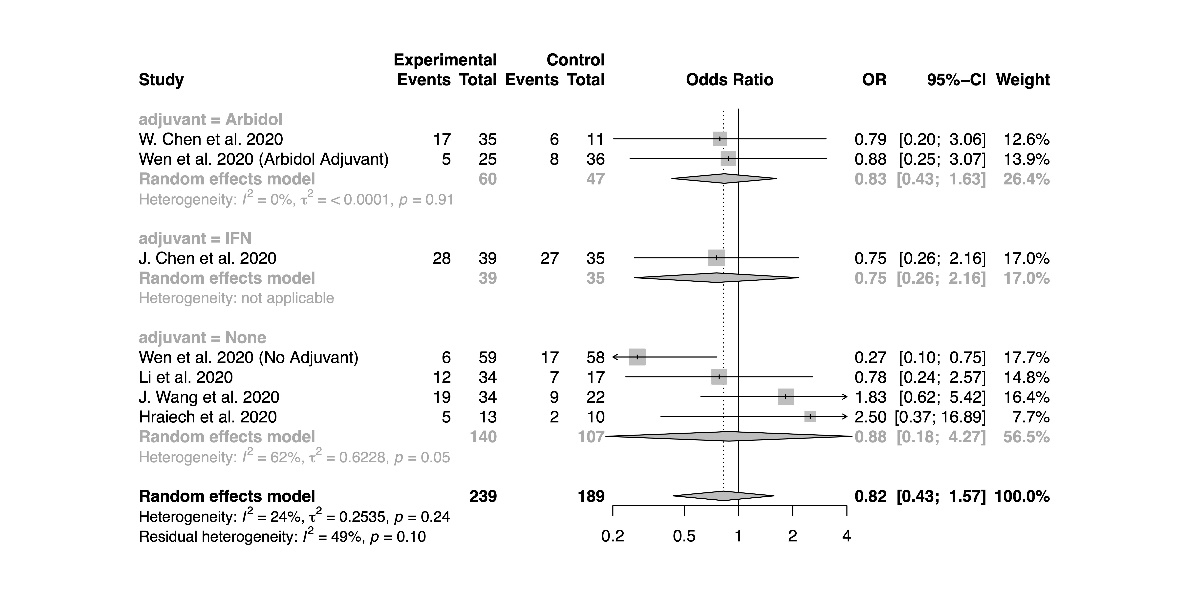** |
| **Figure S19** Forest plot for subgroup analysis by study design, incidence of positive-to-negative conversion of SARS-CoV-2 nucleic acid test at day 7  There is no significant difference between the pooled odds ratio from randomized studies versus non-randomized studies (P=0.87). | **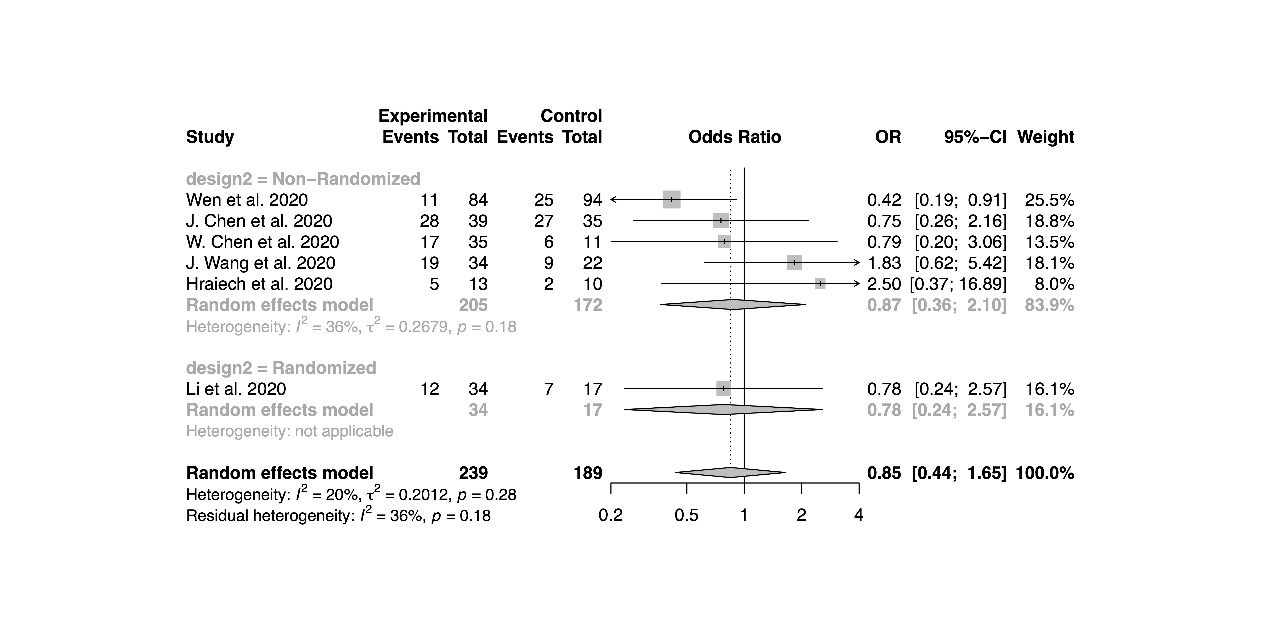** |
| **Figure S20** Forest plot for subgroup analysis by study design, incidence of positive-to-negative conversion of SARS-CoV-2 nucleic acid test at day 14  There is no significant difference between the pooled odds ratio from randomized studies versus non-randomized studies (P=0.97). | **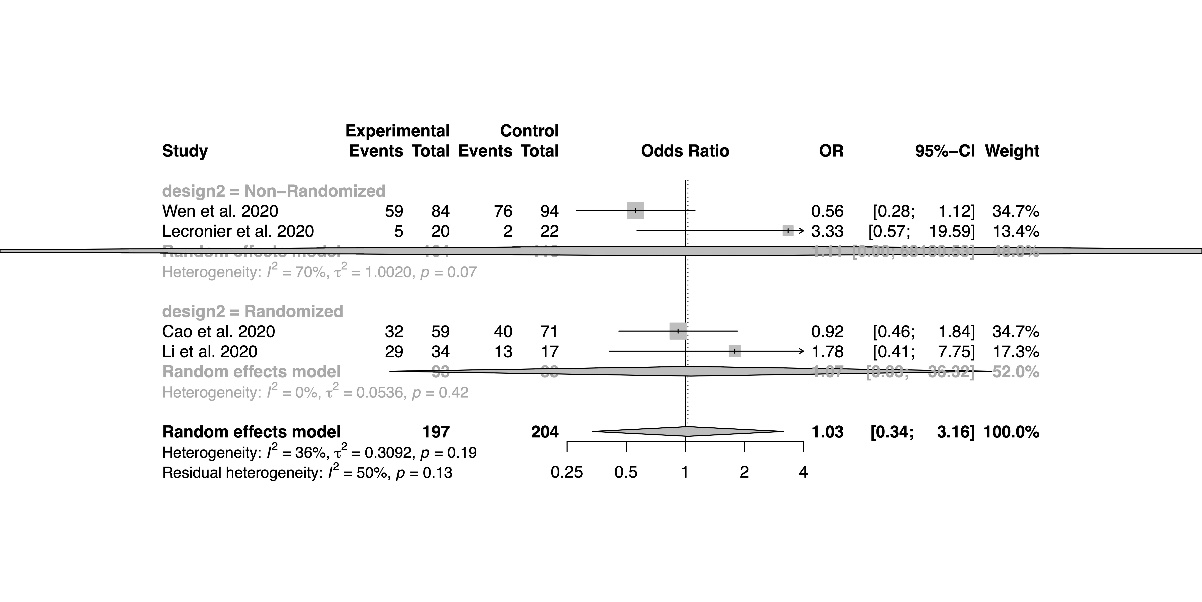** |
| **Figure S21** Forest plot for subgroup analysis by lopinavir/ritonavir regimens, incidence of positive-to-negative conversion of SARS-CoV-2 nucleic acid test at day 7  There is no significant difference between the pooled odds ratio of different regimen subgroups (P=0.24). | **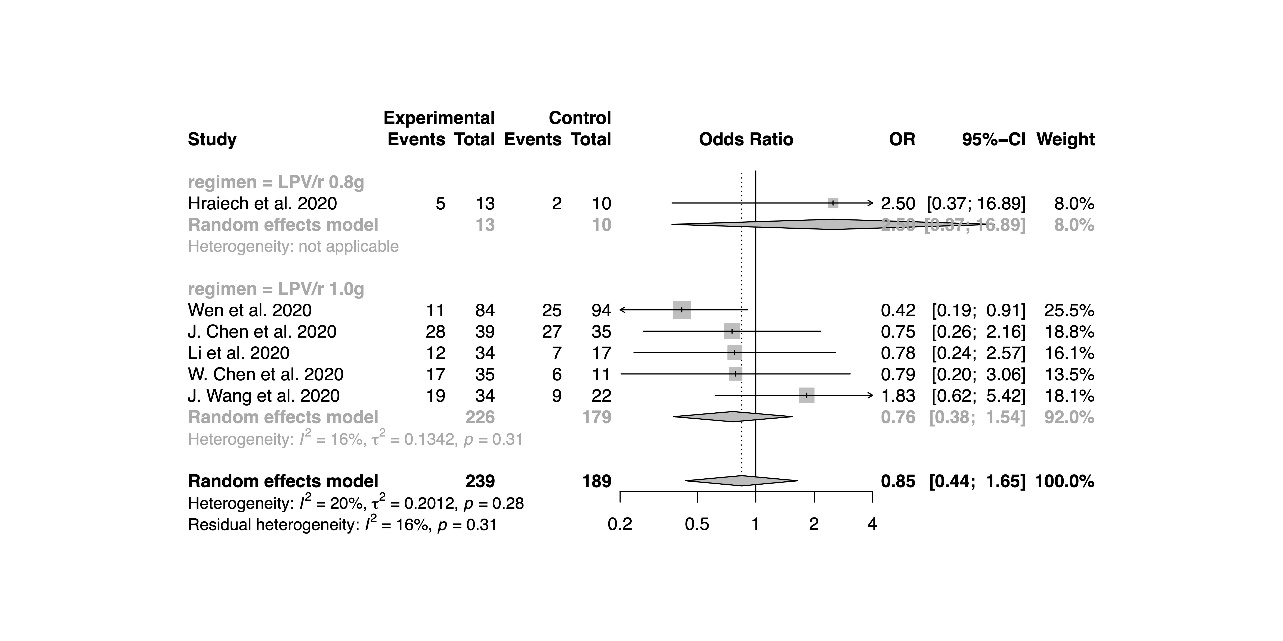** |
| **Figure S22** Forest plot for subgroup analysis by lopinavir/ritonavir regimens, incidence of positive-to-negative conversion of SARS-CoV-2 nucleic acid test at day 14  There is no significant difference between the pooled odds ratio of different regimen subgroups (P=0.14). | **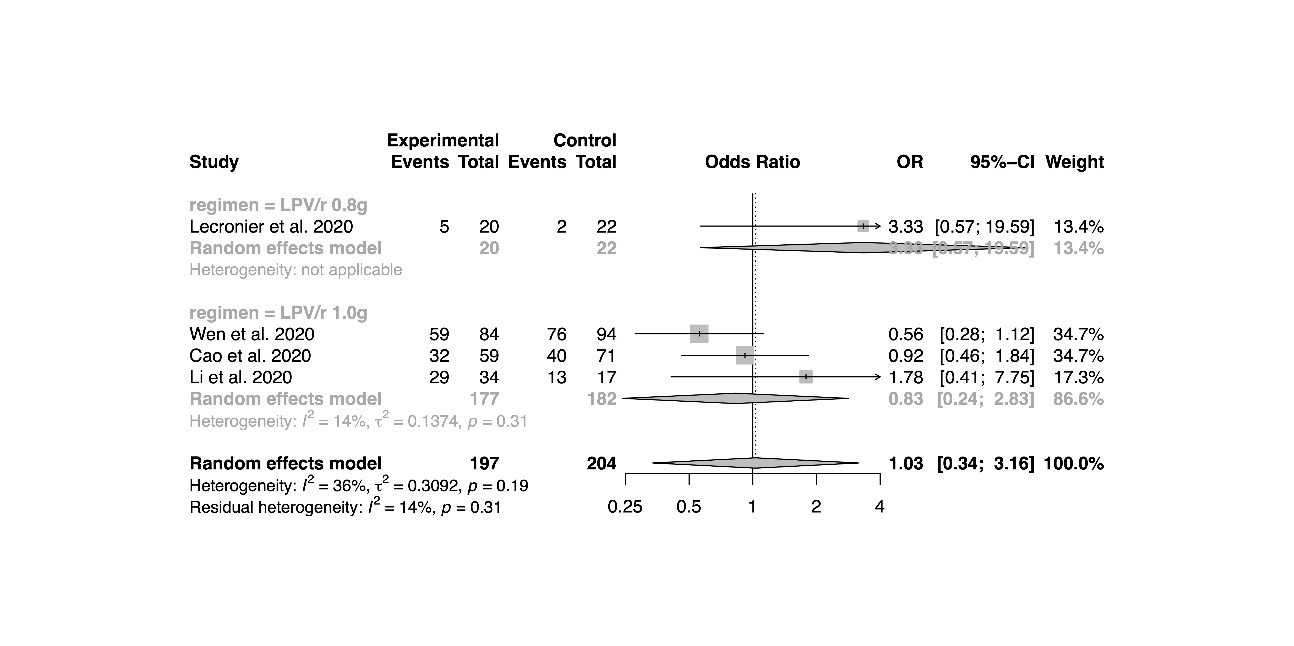** |
| **Figure S23** Bubble plot for meta-regression of proportion of patients with severe disease, incidence of positive-to-negative conversion of SARS-CoV-2 nucleic acid test at day 7  There is no significant correlation between the proportion of patients with severe disease and the treatment effect (P=0.07). | **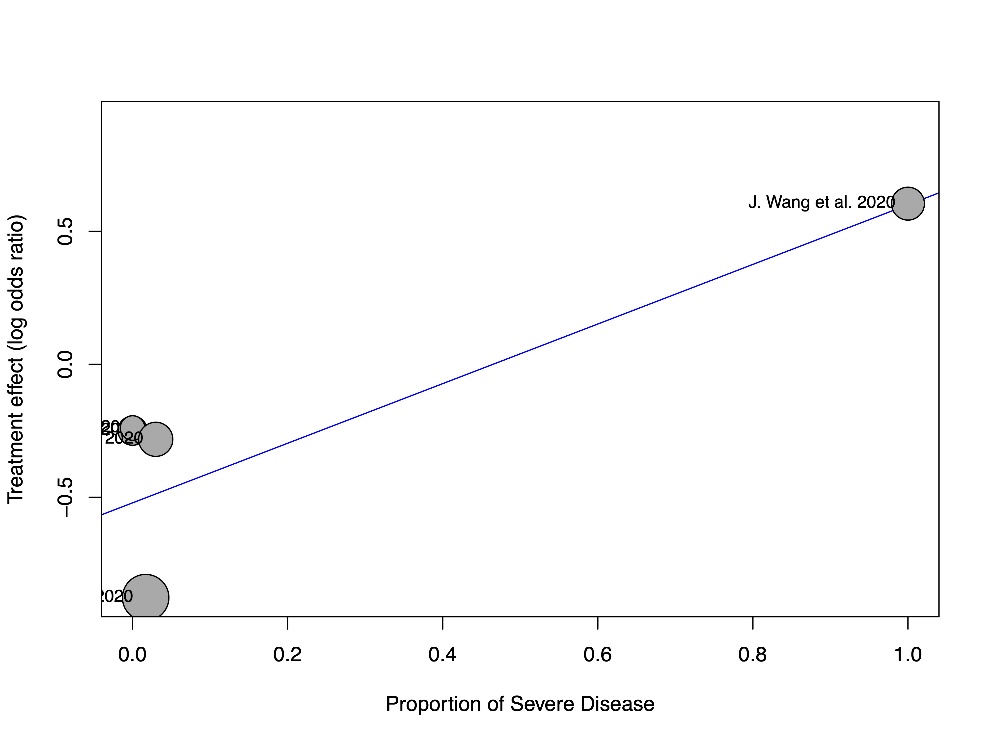** |
| **Figure S24** Bubble plot for meta-regression of proportion of patients with severe disease, incidence of positive-to-negative conversion of SARS-CoV-2 nucleic acid test at day 14  There is no significant correlation between the proportion of patients with severe disease and the treatment effect (P=0.62). | **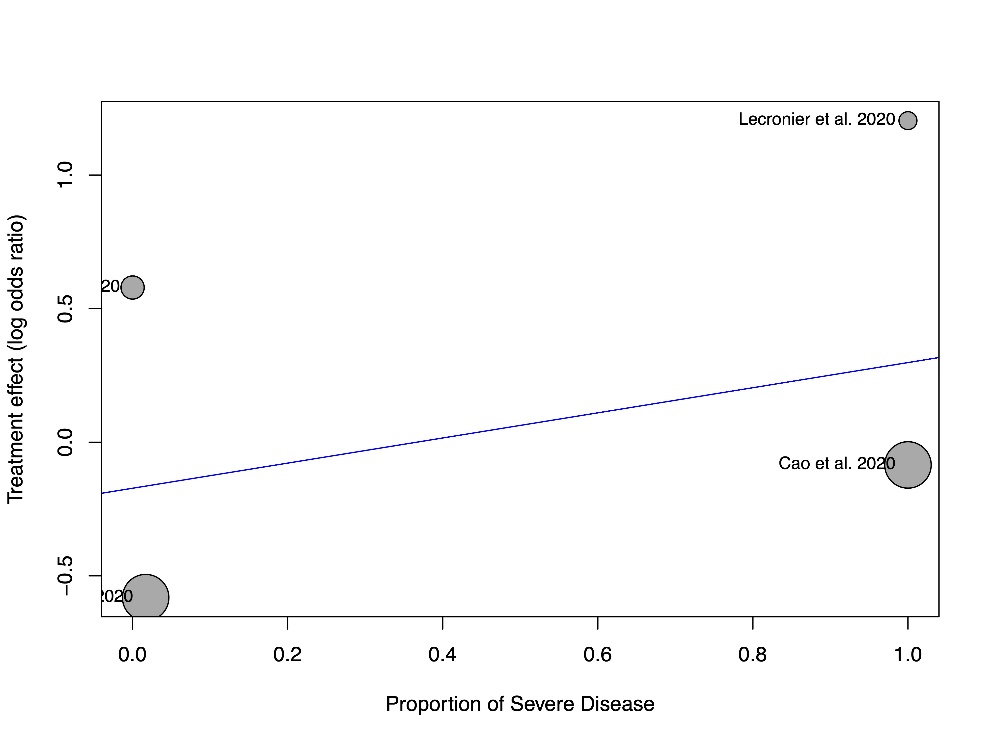** |
| **Figure S25** Funnel plot, incidence of positive-to-negative conversion of SARS-CoV-2 nucleic acid test at day 7  There is no evidence of small study effects based on visual inspection of the funnel plot. | **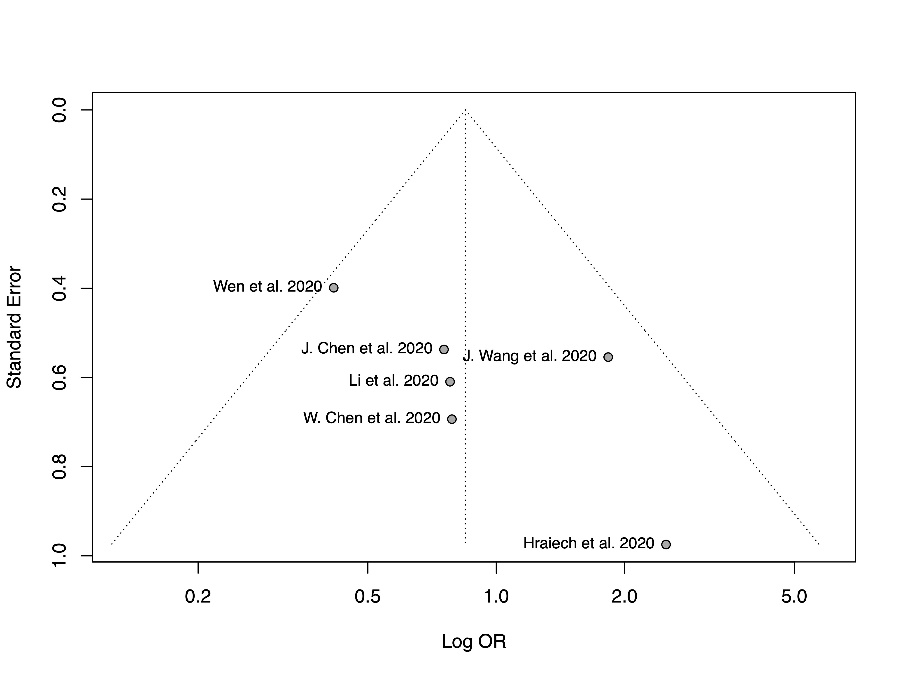** |
| **Figure S26** Funnel plot, incidence of positive-to-negative conversion of SARS-CoV-2 nucleic acid test at day 14  There is no evidence of small study effects based on visual inspection of the funnel plot. | **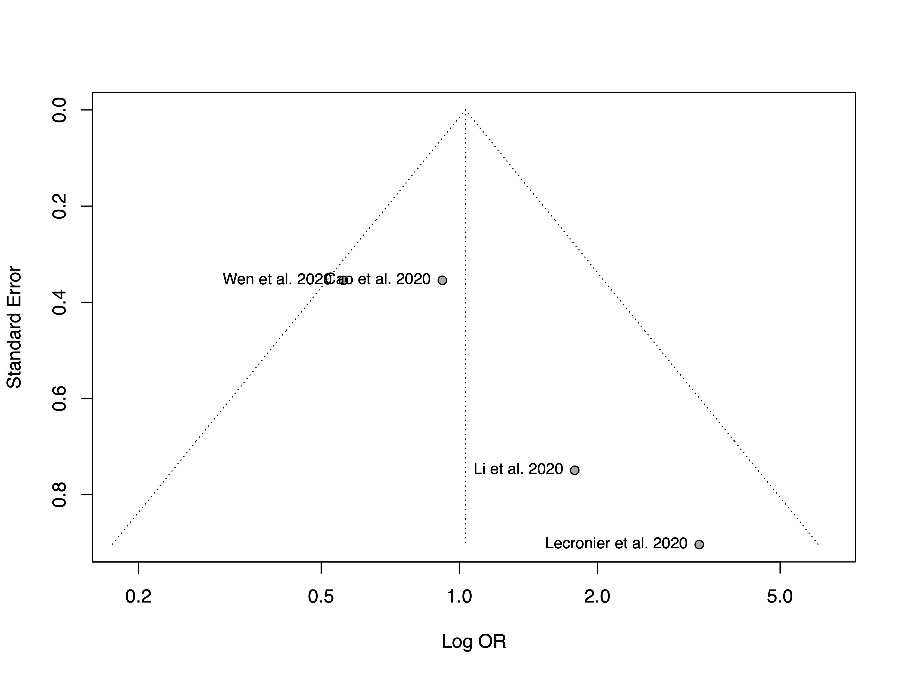** |
| **Figure S27** Forest plot for subgroup analysis by study design, incidence of mechanical ventilation  There is no significant difference between the pooled odds ratio from randomized studies versus non-randomized studies (P=0.60). | **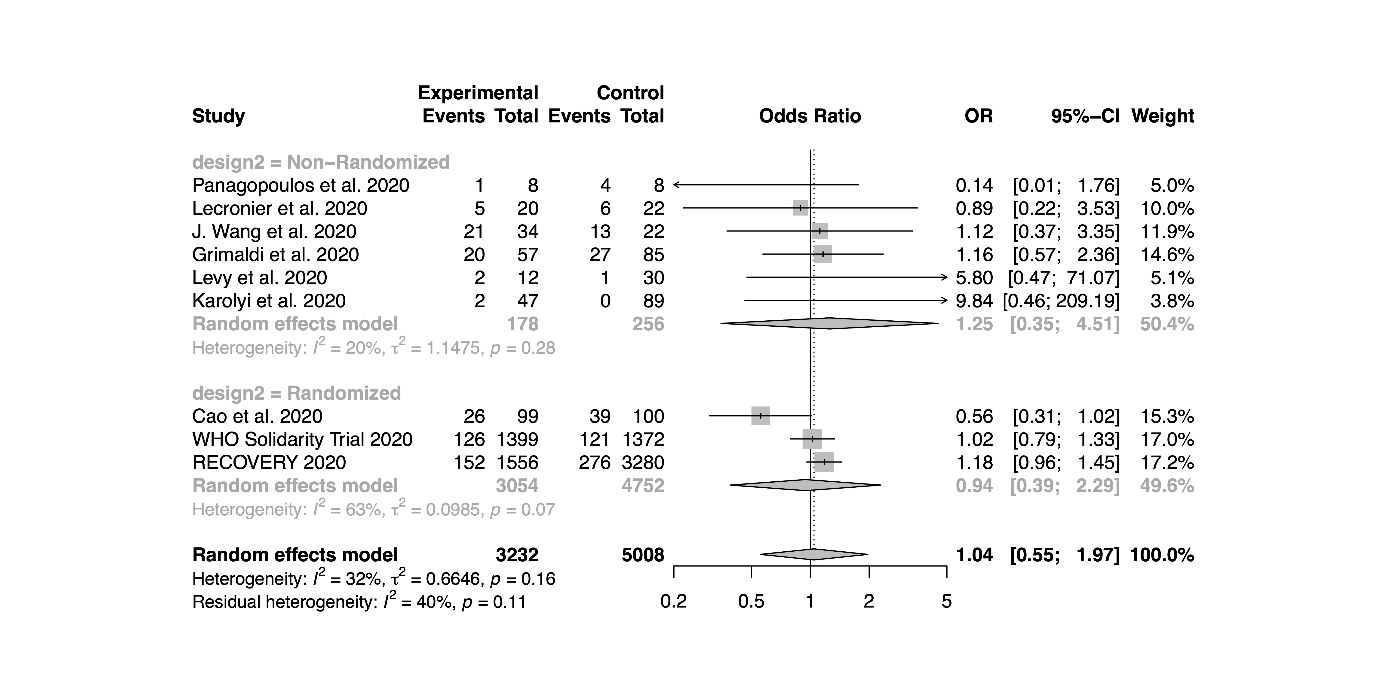** |
| **Figure S28** Forest plot for subgroup analysis by lopinavir/ritonavir regimens, incidence of mechanical ventilation  There is no significant difference between the pooled odds ratio of different regimen subgroups (P=0.71). | **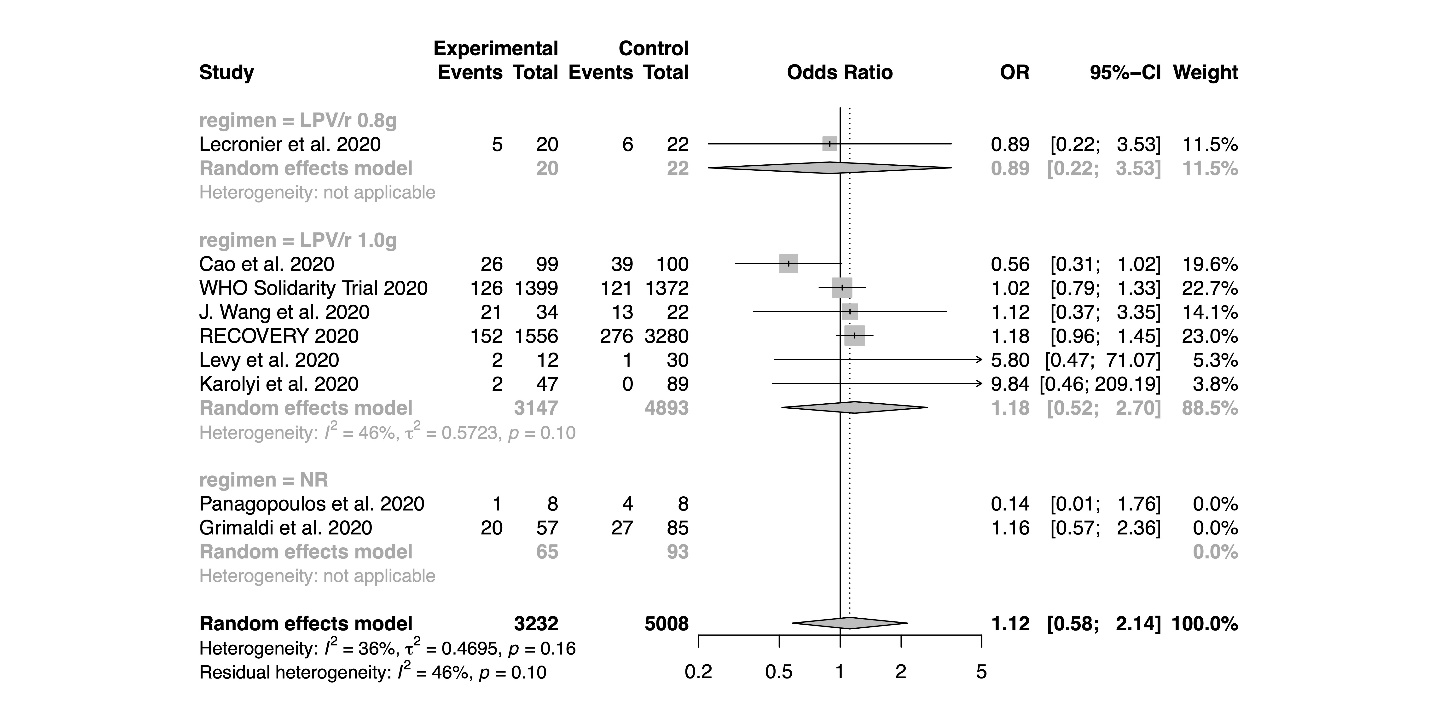** |
| **Figure S29** Forest plot for subgroup analysis by adjuvant therapies, incidence of mechanical ventilation  There is no significant difference between the pooled odds ratio of different adjuvant subgroups (P=0.12). | 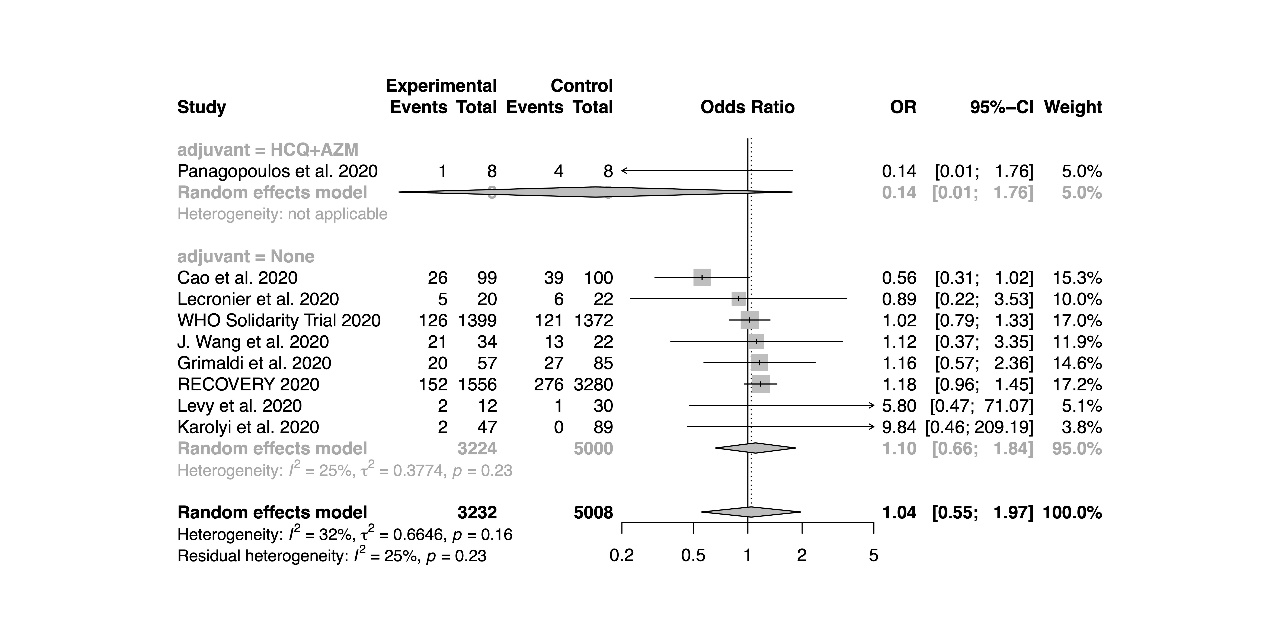 |
| **Figure S30** Funnel plot, incidence of mechanical ventilation  There is no evidence of small study effects based on visual inspection of the funnel plot. | **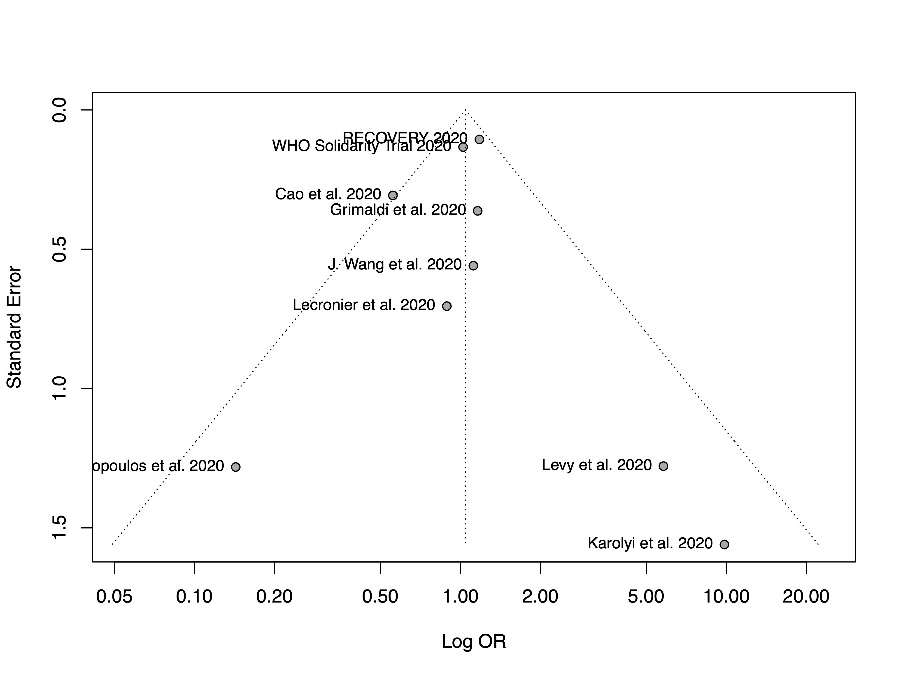** |
| **Figure S31** Forest plot for subgroup analysis by adjuvant therapies, time to body temperature normalization  There is no significant difference between the pooled mean differences of different adjuvant subgroups (P=0.07). | 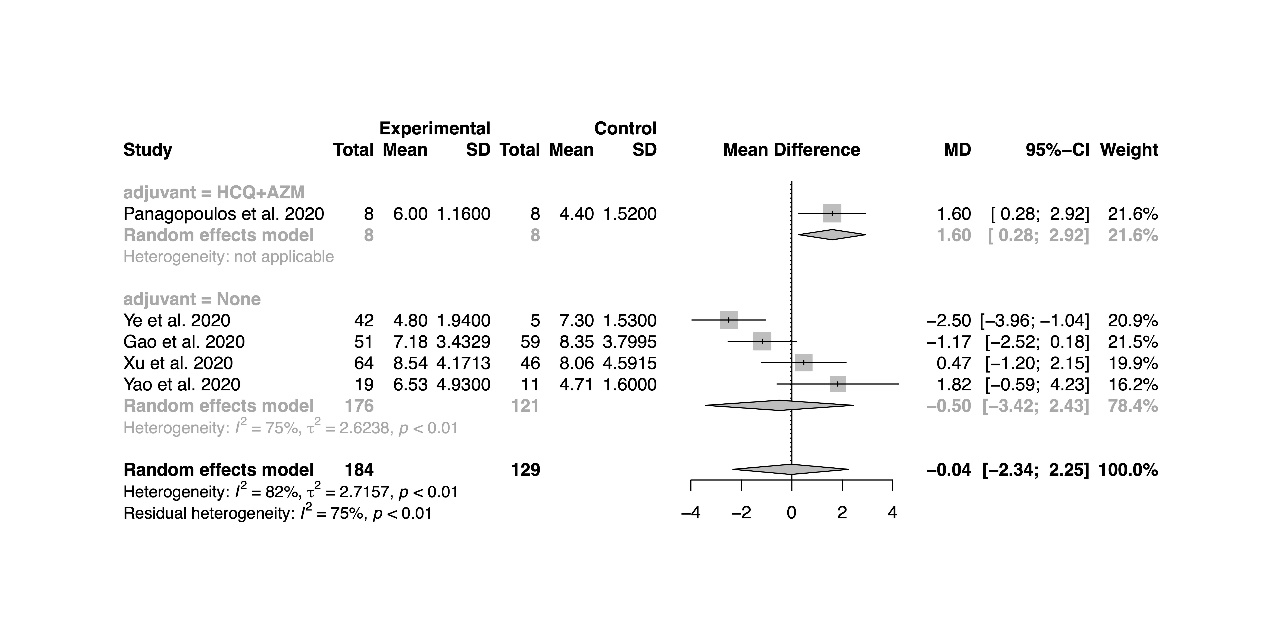 |
| **Figure S32** Forest plot for subgroup analysis by imputation, time to body temperature normalization  There is no significant difference between the pooled mean differences of studies using imputation versus studies that do not require imputation (P=0.68). | **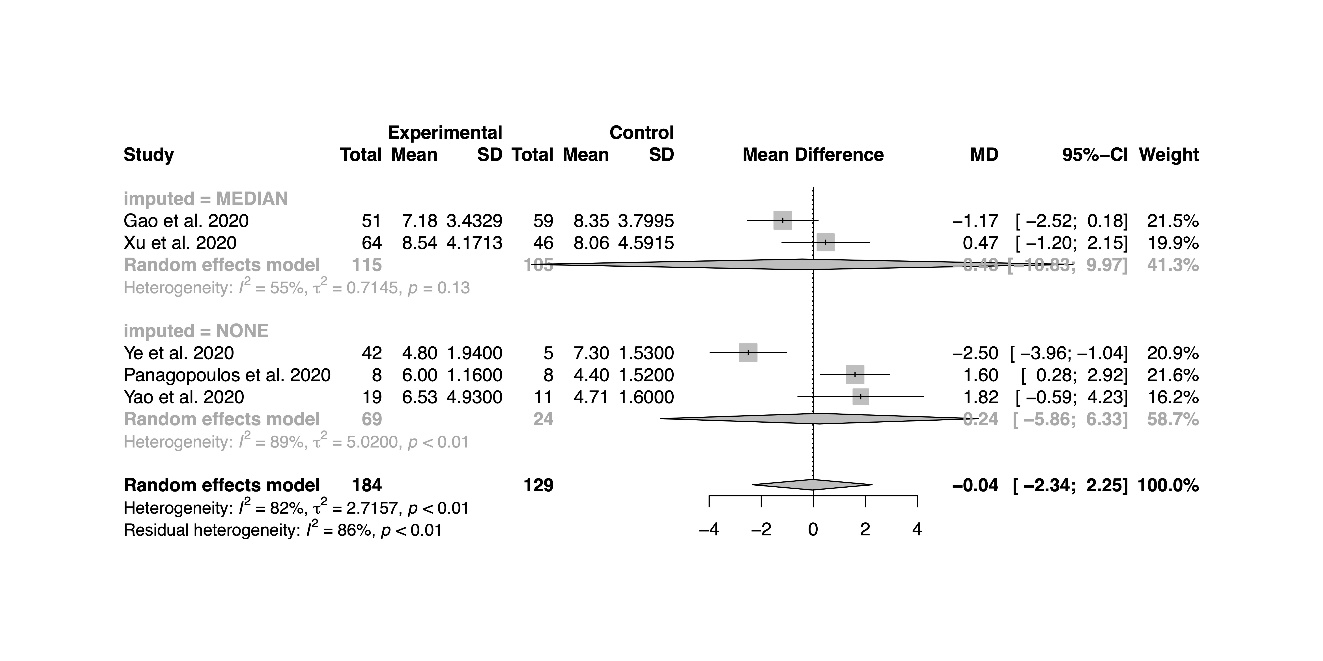** |
| **Figure S33** Bubble plot for meta-regression of proportion of patients with severe disease, time to body temperature normalization  There is no significant correlation between the proportion of patients with severe disease and the treatment effect (P=0.20). | **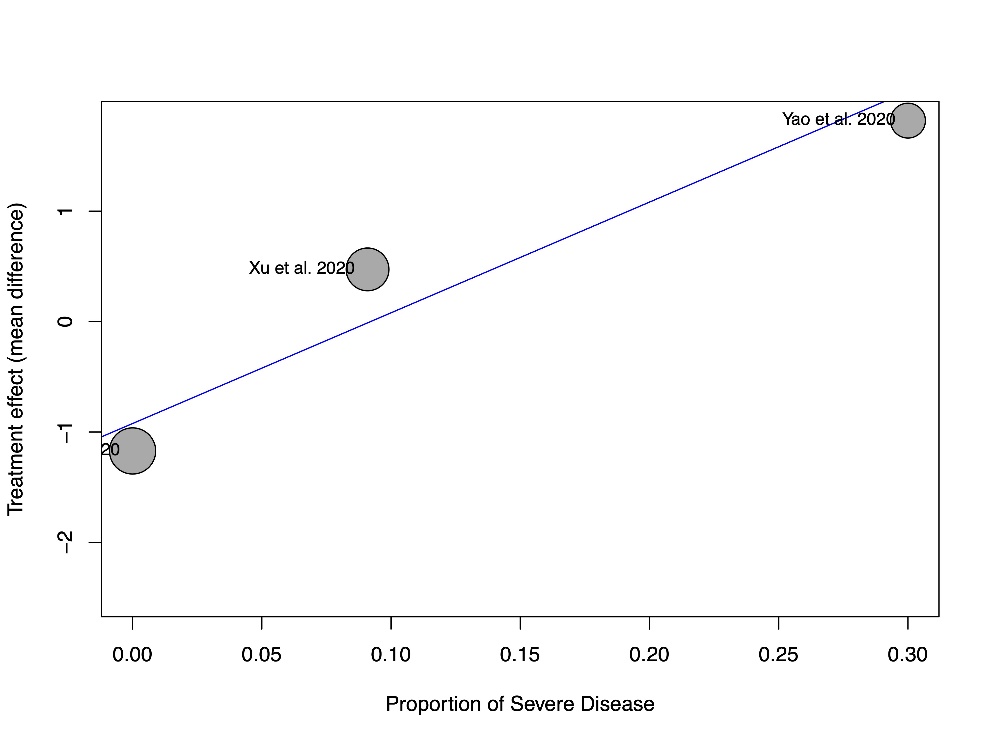** |
| **Figure S34** Funnel plot, time to body temperature normalization  There is no evidence of small study effects based on visual inspection of the funnel plot. | **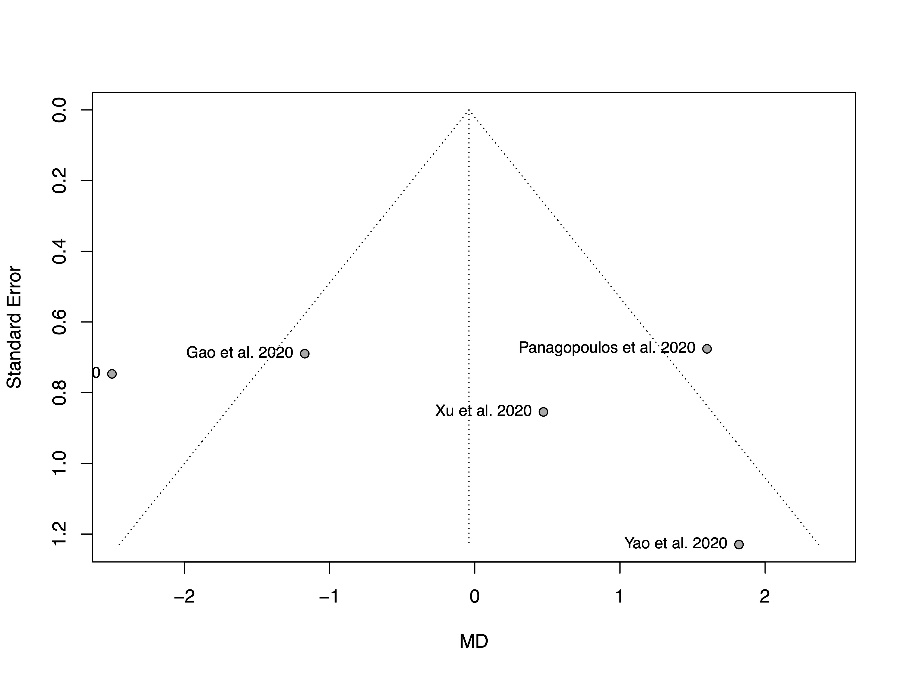** |
| **Figure S35** Forest plot for subgroup analysis by study design, incidence of adverse events  There is no significant difference between the pooled odds ratio from randomized studies versus non-randomized studies (P=0.95). | **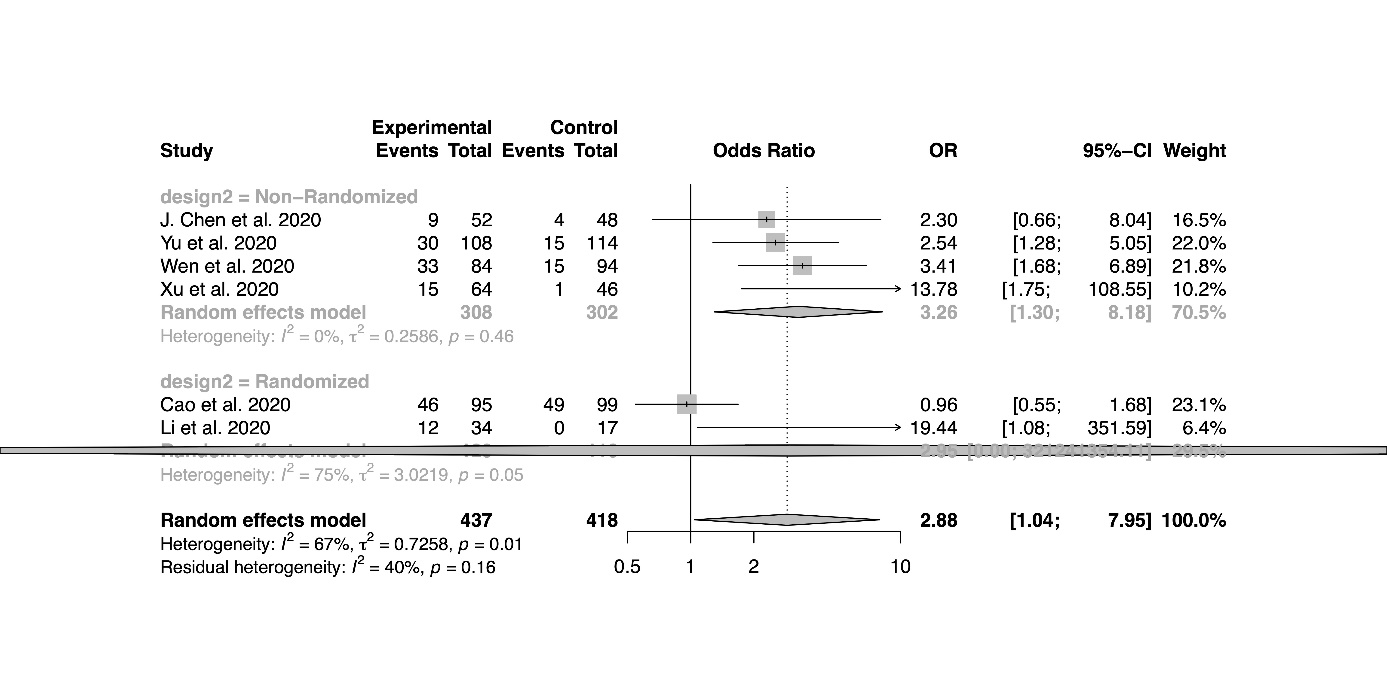** |
| **Figure S36** Forest plot for subgroup analysis by adjuvant therapies, incidence of adverse events  There is no significant difference between the pooled odds ratio of different adjuvant subgroups (P=0.61). | 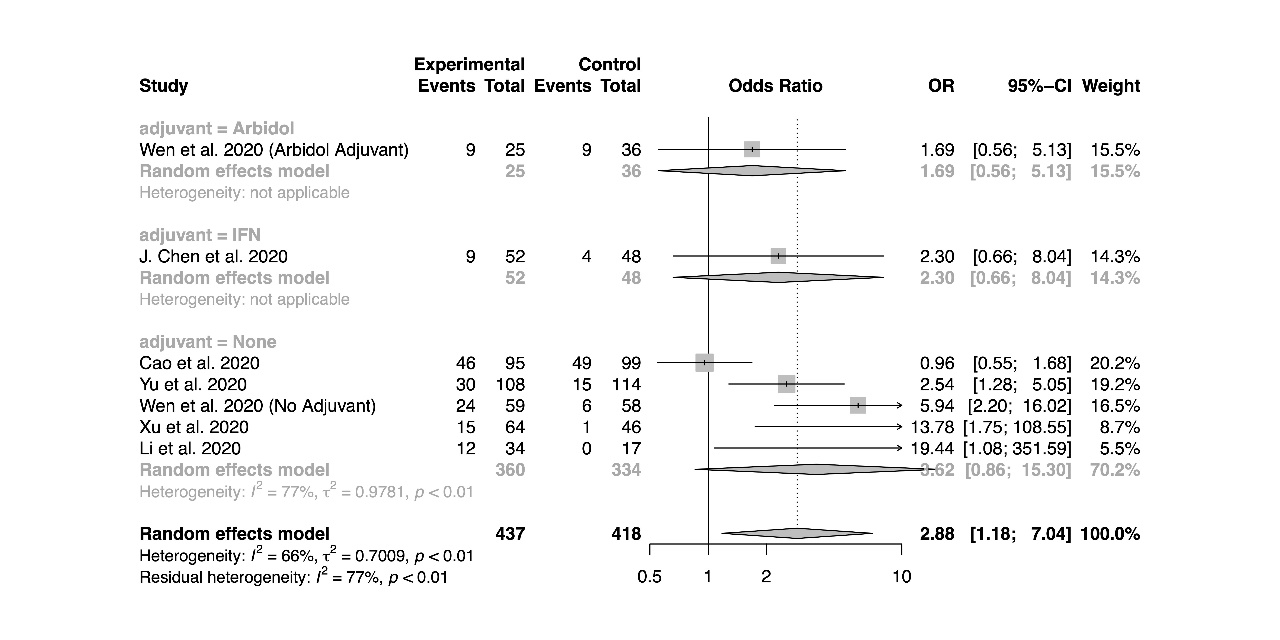 |
| **Figure S37** Bubble plot for meta-regression of proportion of patients with severe disease, incidence of adverse events  There is no significant correlation between the proportion of patients with severe disease and the treatment effect (P=0.11). | **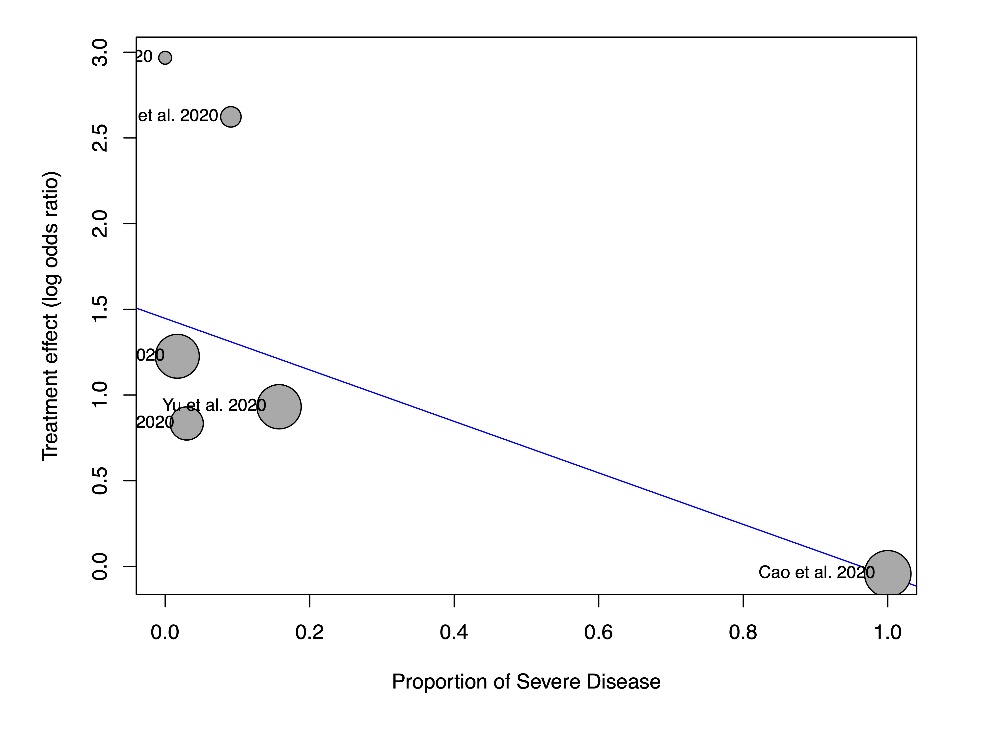** |
| **Figure S38** Funnel plot, incidence of adverse events  There is no evidence of small study effects based on visual inspection of the funnel plot. | **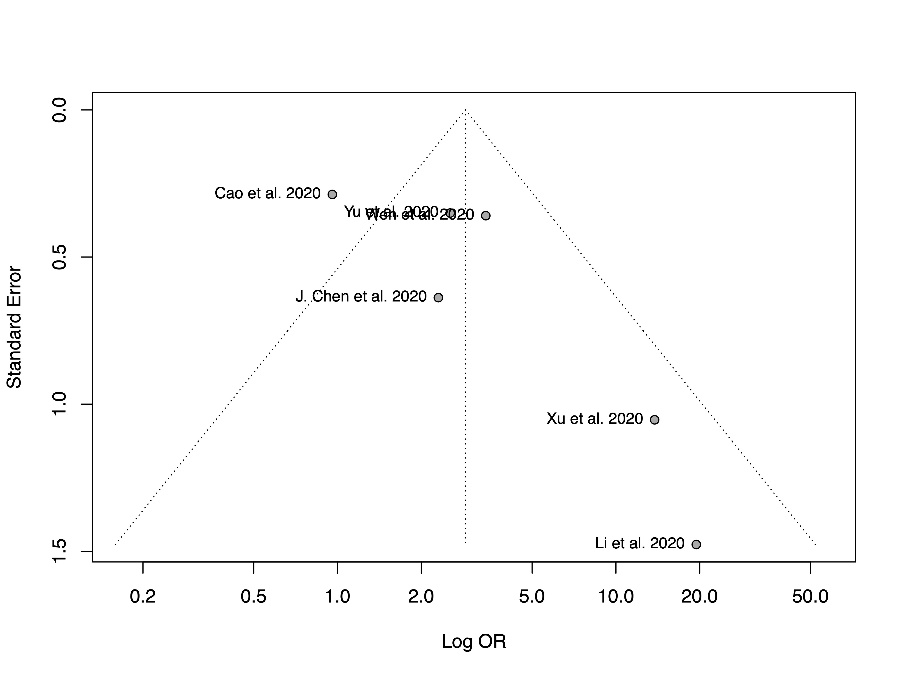** |
